# Evolution of the Antisense Overlap between Genes for Thyroid Hormone Receptor and Rev-erbα and Characterization of an Exonic G-Rich Element That Regulates Splicing of TRα2 mRNA

**DOI:** 10.1371/journal.pone.0137893

**Published:** 2015-09-14

**Authors:** Stephen H. Munroe, Christopher H. Morales, Tessa H. Duyck, Paul D. Waters

**Affiliations:** 1 Department of Biological Sciences, Marquette University, Milwaukee, Wisconsin, United States of America; 2 School of Biotechnology and Biomolecular Sciences, Faculty of Science, UNSW Australia, Sydney, Australia; CNRS UMR7275, FRANCE

## Abstract

The α-thyroid hormone receptor gene (TRα) codes for two functionally distinct proteins: TRα1, the α-thyroid hormone receptor; and TRα2, a non-hormone-binding variant. The final exon of TRα2 mRNA overlaps the 3’ end of Rev-erbα mRNA, which encodes another nuclear receptor on the opposite strand of DNA. To understand the evolution of this antisense overlap, we sequenced these genes and mRNAs in the platypus *Orthorhynchus anatinus*. Despite its strong homology with other mammals, the platypus TRα/Rev-erbα locus lacks elements essential for expression of TRα2. Comparative analysis suggests that alternative splicing of TRα2 mRNA expression evolved in a stepwise fashion before the divergence of eutherian and marsupial mammals. A short G-rich element (G30) located downstream of the alternative 3’splice site of TRα2 mRNA and antisense to the 3’UTR of Rev-erbα plays an important role in regulating TRα2 splicing. G30 is tightly conserved in eutherian mammals, but is absent in marsupials and monotremes. Systematic deletions and substitutions within G30 have dramatically different effects on TRα2 splicing, leading to either its inhibition or its enhancement. Mutations that disrupt one or more clusters of G residues enhance splicing two- to three-fold. These results suggest the G30 sequence can adopt a highly structured conformation, possibly a G-quadruplex, and that it is part of a complex splicing regulatory element which exerts both positive and negative effects on TRα2 expression. Since mutations that strongly enhance splicing *in vivo* have no effect on splicing *in vitro*, it is likely that the regulatory role of G30 is mediated through linkage of transcription and splicing.

## Introduction

Nuclear receptors (NRs) form a large superfamily of structurally related ligand-activated transcription factors [1]. Almost fifty NRs are encoded in the genomes of mammals. Among these, two genes, those for Rev-erbα (NR1D1) and the α-thyroid hormone receptor (TRα, also NR1A1 and THRA), share a unique relationship in that they are closely linked and transcribed from opposite strands of the DNA in a convergent direction [2]. In some mammals the final exons of Rev-erbα and a variant TRα receptor share an antisense overlap [2,3]. The unusual organization of the TRα/Rev-erbα locus raises interesting questions concerning the evolution and expression of overlapping genes. Several lines of evidence suggest that the overlap between these two genes plays a role in regulating the expression of their mRNAs [2,4–8]. However, the mechanism and physiological significance of this regulation remain unclear.

Rev-erbα and TRα play critical roles in regulating development and metabolism in response to a variety of autonomous and environmental cues [9–11]. As a core component of the molecular clock, Rev-erbα expression displays strong circadian rhythmicity, and it in turn regulates the expression of hundreds of downstream genes [9,12,13]. The canonical form of the TRα receptor, TRα1, mediates the activity of thyroid hormone. TRα1 promotes transcription of hormone-responsive genes in the presence of thyroid hormone and represses them in its absence [14]. Most mammals also express a non-hormone-binding isoform, TRα2, from an alternatively spliced mRNA that overlaps the final exon of Rev-erbα [3]. The unique C-terminal domain of TRα2 lacks a functional hormone-binding domain. Thus, TRα2 functions as a constitutive, dominant-negative repressor of TRα1-mediated hormone activity [15–17]. Although questions remain concerning the physiological significance of this interaction [10,11,14], the tissue- and stage-specific expression of TRα1 and TRα2 is well established [18,19]. The two proteins also differ in their location within the cell: TRα1 is predominantly nuclear whereas TRα2 has a substantial cytoplasmic component [20]. The latter observations suggest that TRα2 may have additional functions that further distinguish it from TRα1.

The evolutionary history of these genes provides important clues regarding the functional differentiation of these regulatory proteins. In most, if not all, vertebrates, two paralogs of TRα and Rev-erbα are also present: TRβ (NR1A2) and Rev-erbβ (NR1D2). In amphibians, reptiles, birds and mammals these genes are also found at adjacent, convergently transcribed sites, suggesting an ancient origin for this organization. However, TRβ and Rev-erbβ are usually more widely spaced than TRα and Rev-erbα with no evidence of transcriptional overlap. In fact, the antisense overlap associated with alternative splicing of TRα2 is observed only in mammals. A previous study from this laboratory also suggests that TRα2 is not expressed in marsupials even though most core splice site elements required for TRα2 expression are present [3].

To better understand the origins of the antisense overlap between Rev-erbα and TRα2, we have extended our analysis of this locus to the platypus, *Ornithorhynchus anatinus*, a unique representative of one of two surviving genera of monotremes, which comprise the earliest branching of extant mammals. Recent fossil evidence suggests that monotremes diverged from other mammals in the late Triassic, more than 208 Myr before present [21,22],while later times are supported by molecular analysis [23,24]. There is general agreement that monotremes diverged prior to the split between marsupial and eutherian mammals at a time roughly halfway between the divergence of mammals and reptiles and the major radiation of modern eutherian mammals [24–27]. Consistent with this chronology, monotremes are the only egg-laying mammals and display numerous other characteristics that support their affinity with reptiles and birds [28].

In this study we observe both striking differences and notable similarities in comparing the sequences of the TRα/Rev-erbα genes in platypus and other vertebrate species. First, the platypus TRα/Rev-erbα locus displays many features characteristic of other mammals that are absent in non-mammalian vertebrates, with the notable exception of core splice site elements for TRα2 mRNA. Second, well-conserved elements in the 3’ UTR of Rev-erbα suggest that this mRNA has multiple poly(A) sites common to all three branches of mammalian evolution. Third, comparative analysis suggests that the origins of alternative splicing of a TRα2-like mRNA pre-date the marsupial/eutherian divergence.

Finally, we describe the role of a G-rich element that is located near the 3’ splice site (ss) in the final exon of TRα2 mRNA and antisense to the 3’ UTR of Rev-erbα mRNA at a site adjacent to the stop codon. This element, G30, strongly restricts TRα2 splicing. Deletions and point mutations in any one of several clusters of G residues greatly enhance TRα2 mRNA splicing. On the other hand, mutations targeted to a central region strongly inhibit splicing. The G30 element is almost totally conserved in eutherian mammals but absent in marsupials and platypus. The enhancement of TRα2 splicing seen *in vivo* following deletion or substitution within the G clusters of G30 is not observed when pre-mRNAs bearing the same mutations are spliced *in vitro*. These results suggest that balanced expression of TRα1 and TRα2 requires multiple closely spaced G-clusters that may form a G-quadruplex structure that restricts TRα2 splicing *in vivo*.

Taken together, our results suggest that alternative splicing of TRα2 evolved by stepwise exaptation of existing elements within and adjacent to the coding sequences for TRα1 and Rev-erbα. Multiple mechanisms, including alternative polyadenylation and splicing, coordinately regulate expression of these closely linked genes. Our results also suggest a novel mechanism in which a G-rich structure plays a role in linking transcription and mRNA splicing.

## Results

### Sequencing the platypus TRα/Rev-erbα locus

**Fig 1:**
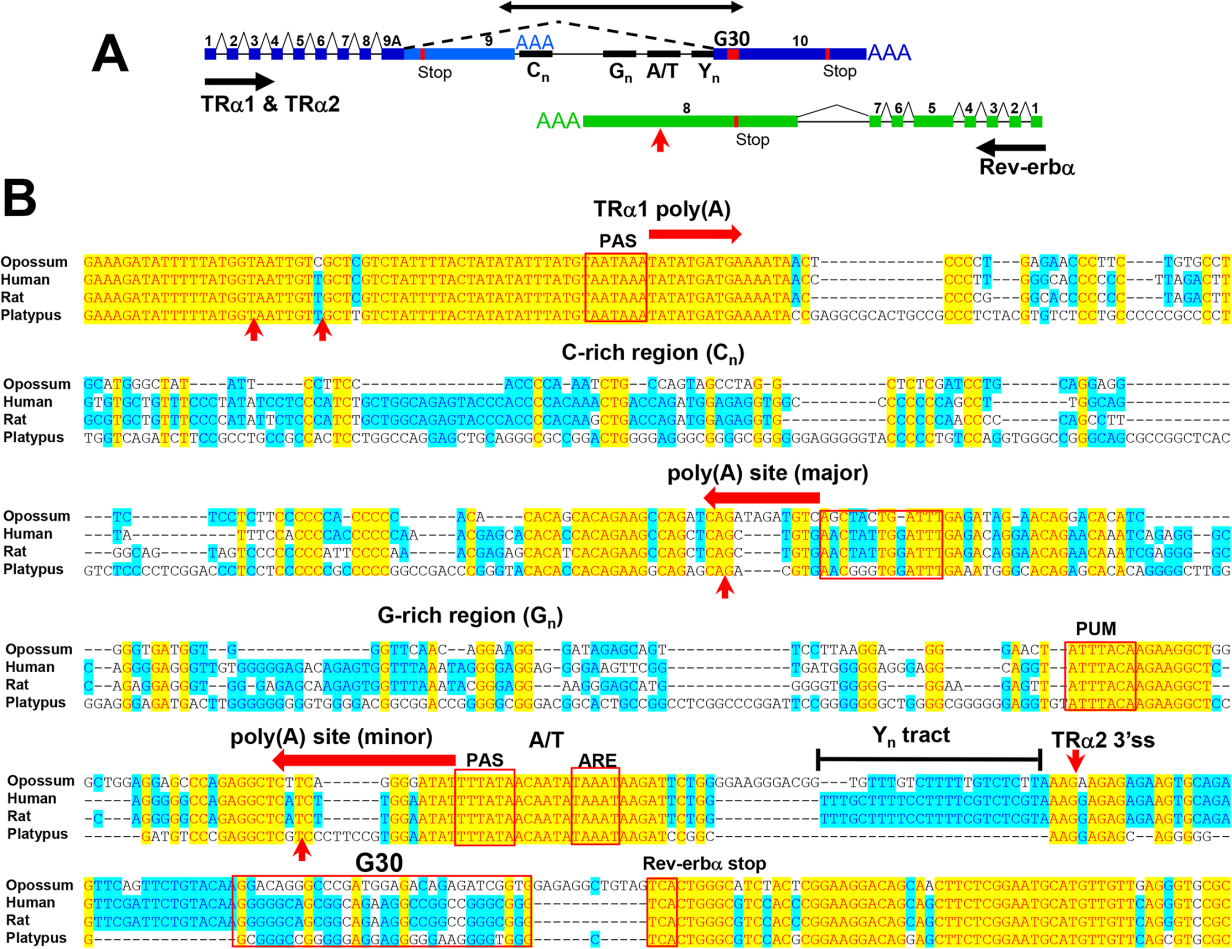
Structure of the TRα/Rev-erbα locus. **(A**) Schematic representation of the conserved exon structure of TRα1, TRα2 and Rev-erbα mRNAs in eutherian mammals. Exons and introns are indicated by thick boxes and horizontal lines, respectively, with exon numbers indicated above. Horizontal arrows indicate direction of transcription; AAA represents major poly(A) sites. TRα1 mRNA includes exons 1–9. TRα2 mRNA includes exons 1–8, 9A and 10. Constitutive splicing is indicated by the angled solid lines, and the angled dotted line represents the alternative splicing of TRα2 mRNA. Thin boxes labeled C_n_, G_n_, A/T, Y_n_ and G30 represent conserved regions enriched in the indicated nucleotides as described in text; the vertical red arrow indicates the minor poly(A) site for Rev-erbα; and the double-headed arrow indicates the region shown in Panel B. The stop codon for each mRNA is indicated with a vertical red line. (**B**) Alignment of sequences from four mammals extending from the 3’ end of TRα1 mRNA (on the strand shown here) to the 3’ portion of exon 8 of Rev-erbα mRNA on the complementary strand. Yellow shading indicates identical residues in all aligned sequences; blue shading indicates identical residues in at least half of the aligned sequences. Red boxes delineate conserved sequence elements: the hexanucleotide polyadenylation signals (PAS), the non-canonical AT-rich sequence upstream of the major Rev-erbα poly(A) site (A/T), the consensus PUF protein-binding site (PUM) in Rev-erbα mRNA, and an AU-rich element in Rev-erbα (ARE). TRα1 and Rev-erbα poly(A) sites in rat and human are marked with large horizontal red arrows, the TRα2 3’ss with a downward vertical red arrow and poly(A) sites in platypus Rev-erbα mRNA with upward vertical red arrows.

The structure of the TRα/Rev-erbα locus in eutherian mammals is shown in Fig 1A. The sequence of the 3’ ends of these genes is nearly invariant in eutherian mammals and surprisingly well conserved in marsupials despite the absence of TRα2 expression (Fig 1B) [3]. To better understand the evolution of this locus, we sequenced a region extending across the 3’ ends of both genes that is missing from the draft assembly of the platypus genome [28]. This approach yielded a continuous 7 kb sequence extending from exon 4 of TRα to exon 6 of the Rev-erbα gene (GenBank accession KR028100). This sequence was then used together with PCR sequencing to assemble the complete coding sequences of the platypus TRα1 and Rev-erbα mRNAs from high throughput reads of the platypus transcriptome [29]. The final set of genomic and mRNA sequences included multiple overlaps that were used to cross-check and validate the final assemblies. For details see Material and Methods and S1 Fig.

As expected, the amino acid sequence of platypus Rev-erbα is more similar to that of mammals (78–80% identity) than to that of other vertebrates, including frog, lizard, turtle, chick and falcon (68–73% identity; S2A Fig and S2C Fig). The amino acid sequence of platypus TRα1 is more highly conserved, but displays little variation (93–94% identity) in its similarity to that of other mammals, birds and reptiles (S3A and S3C Fig). The lower conservation of Rev-erbα reflects the presence of a poorly conserved hinge region connecting the conserved DNA-binding domain with the C-terminal ligand-binding domain (LBD). The LBD of the platypus Rev-erbα, comprising 192 amino acid residues, is 95–96% identical to that of other mammals and 81–91% identical to that of other vertebrates in an ungapped alignment (S2A Fig). The final 66 amino acids of the Rev-erbα LBD are encoded by exon 8, which is antisense to the final exon of TRα2 in eutherian mammals. This sequence in platypus differs from that of rat and human at only three sites, each of which also varies in alignment with reptiles, birds and frog (S2A Fig).

A somewhat different perspective emerges in comparing the underlying nucleotide sequence of Rev-erbα mRNA (S2B and S2D Fig). The 200 nt coding sequence in exon 8 in platypus is slightly more similar to that of eutherian mammals (88–89% identity), birds (87%) and turtle (87%) than to that of marsupials (82–85%). These similarities extend throughout the nucleotide sequence of the LBD of Rev-erbα (S3 Fig). They are also reflected in the coding potential of the antisense strand, which corresponds to the eutherian TRα2 sequence (S4 Fig). Interestingly, the predicted amino acid sequence on the antisense strand of platypus Rev-erbα exon 8 more closely resembles the eutherian consensus than any of the marsupial sequences (S4B Fig). Furthermore, the platypus sequence also lacks the multiple stop codons present in the corresponding antisense marsupial Rev-erbα sequences (see also Fig 2 in [3]).

### Conservation of sequence motifs within the TRα2/Rev-erbα overlap region

A sequence alignment that extends from the 3’ end of the TRα1 mRNA through to the final exon of Rev-erbα is shown in Fig 1B. This region is framed by two exceptionally well-conserved regions: the 3’ end of TRα1 and the bidirectional coding sequence for Rev-erbα and TRα2. The intervening region displays many shorter conserved motifs that vary in their level of conservation and their nucleotide composition. These motifs reflect the presence of multiple elements important for transcriptional termination and post-transcriptional processing of both genes (Fig 1B; sequences keyed in Fig 1A).

Between the 3’ end of the TRα1 mRNA and the 3’ splice site for TRα2, two broad regions of conservation are apparent (Fig 1B). One corresponds to the major Rev-erbα poly(A) site; the other is comprised of a series of AT-rich motifs. On either side of the major Rev-erbα poly(A) site are two regions (labeled C_n_ and G_n_) that are enriched in multiple clusters of three or more C and G residues, respectively. Although the specific sequences of C_n_ and G_n_ vary among the major mammalian groups, to some extent they all display a skewed distribution of C and G clusters. In eutherian mammals the C_n_ region between the TRα1 and Rev-erbα poly(A) sites includes almost no clusters of three or more G residues. Conversely, the G_n_ region in eutherian mammals includes no clusters of C residues (Fig 1B and S5 Fig). Clusters (or runs) of G residues are often associated with termination and pause sites downstream of the 3’ end of mRNA [30,31], and such a function seems likely here. On the opposite (i.e. Rev-erbα) strand, antisense to that in Fig 1B, the C_n_ region comprises multiple G-runs that lie directly downstream of the major Rev-erbα poly(A) site. On the TRα strand the G_n_ region is 200–300 nt downstream of the TRα1 poly(A) site.

Interestingly, similar G and C clusters are present in the genomes of birds, which have a similar close spacing of TRα and Rev-erbα genes (S5A Fig). The skewed distribution of clusters of G and C residues evident in the C_n_ and G_n_ regions of marsupial and eutherian mammals is often disrupted in bird sequences. In this respect the platypus sequence most closely resembles that in birds. For example, in both platypus and birds, multiple adjacent G-clusters interrupt the C_n_ region, and C-clusters interrupt the G_n_ region. However, other elements that are conserved among mammals are absent in birds, including the AT-rich sequences associated with the Rev-erbα poly(A) sites.

The exon-exon overlap between TRα2 and Rev-erbα can be subdivided into two regions: the bidirectional coding sequence (truncated at the bottom of Fig 1B) and a region of approximately 60 bp that lies between the TRα2 3’ss and the Rev-erbα stop codon. Within the latter region is a G-rich segment of about 30 nt, designated G30 (labeled box in Fig 1B). The sequence of G30 is notable in that it is nearly invariant among eutherian mammals but very different from the corresponding sequence in marsupials. (Fig 1B and S5B Fig). The platypus sequence aligning with G30—that is, the region adjacent to the Rev-erbα stop codon—is also G-rich (and poor in T residues) but aside from this similarity in base composition it lacks specific resemblance to the G30 sequence in eutherian mammals (or to the corresponding sequence in marsupials; Fig 1B and S5B Fig). However, G-rich regions containing multiple clusters of G-residues similar to those of platypus are found in birds and some reptiles (S5B Fig).

Finally, sequences upstream of the TRα2 3’ss, which include the polypyrimidine tract (Y_n_) essential for 3’splice site activity, are missing in platypus (Fig 1B). In fact, a region of approximately 60 nt between the AT-rich region and G30, which is highly conserved in eutherian and marsupial mammals [3], is completely absent in platypus.

### Evolution of the 5’ splice site for TRα2 mRNA

**Fig 2:**
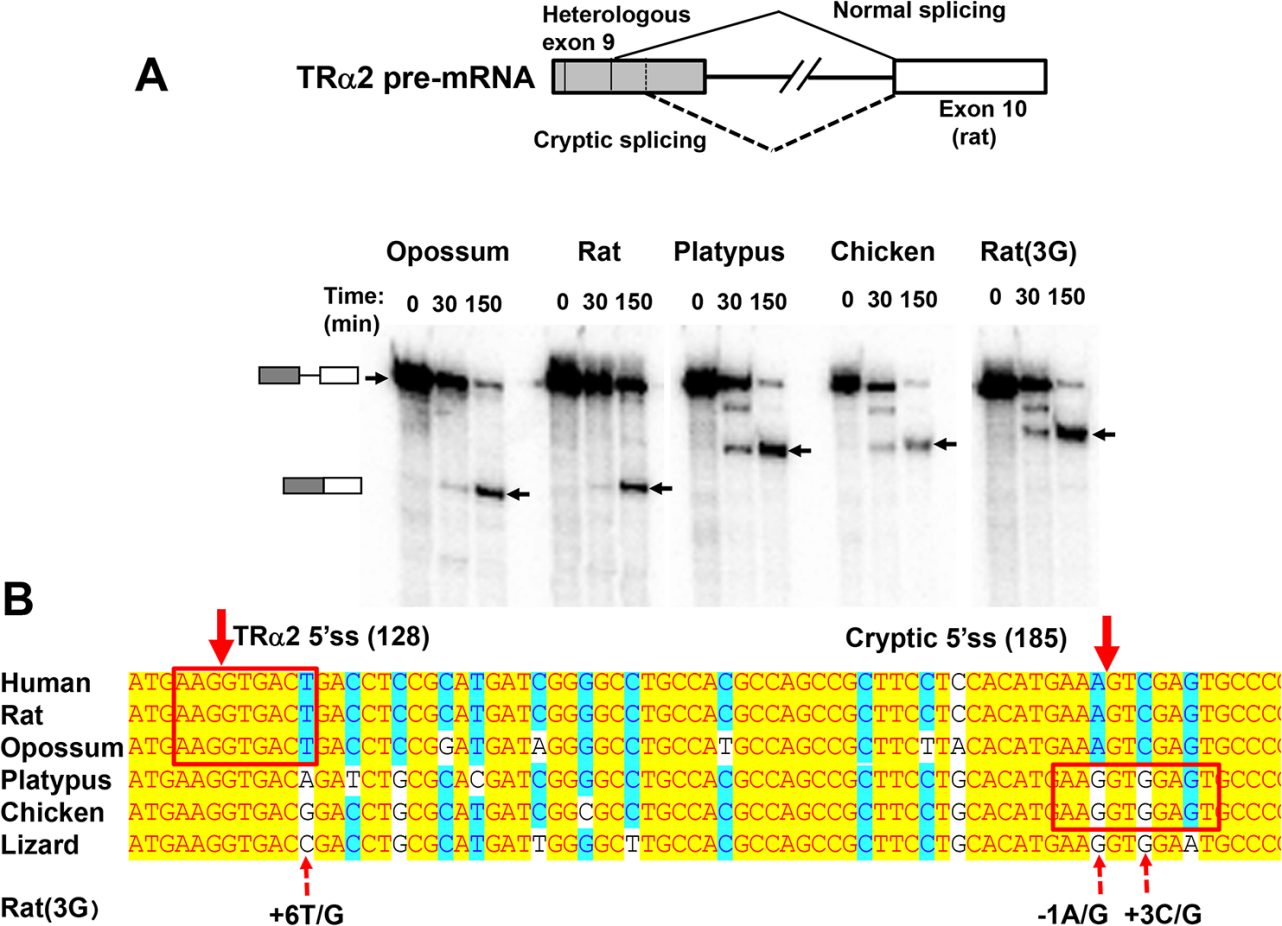
Evolution of the alternative 5’ss in exon 9 of TRα1/TRα2 mRNA. (**A)** Splicing of chimeric pre-mRNAs *in vitro*. Structure of pre-mRNA constructs, indicated on diagram at top, includes exon 9 sequences (shaded box) from opossum, rat, platypus or chicken together with a portion of intron 9 and exon 10 from rat (open box). Rat(3G) is a construct in which three G residues are substituted in the rat pre-mRNA. Labeled pre-mRNAs were incubated with nuclear extract for the times indicated and analyzed by electrophoresis as shown on the autoradiogram. Arrows indicate unspliced pre-mRNA (top) and spliced mRNA, as shown by cartoons at left. Spliced mRNAs were confirmed by RT-PCR sequencing. Note: both rat pre-mRNAs include an additional 13 nt of vector sequence at their 5’ end that results in slower migration relative to the chimeric pre-mRNAs. **(B)** Alignment of sequences adjacent to the 5’ splice site of TRα2 in exon 9. Shading is similar to that in Fig 1B. Normal and cryptic 5’ splice sites are indicated with solid arrows at top with positions relative to the 3’ss for exon 9 given in parentheses. Consensus splice site sequences are boxed. Dotted arrows at bottom indicate residues within the normal or cryptic 5’ss that are conserved in all eutherian and marsupial species. Each of these residues, identified by their position relative to the 5’ss, is replaced with G in the Rat(3G) construct. Human and lizard sequences are shown for comparative purposes.

The absence of sequence homologous to the 3’ss of the TRα2 exon 10 in platypus strongly suggests that platypus, like marsupials, does not express TRα2. In addition to the absence of a 3’ss for this exon, sequences homologous to the 5’ss of TRα2 in platypus more closely resemble those of non-mammalian vertebrates than those of marsupials or eutherian mammals. Specifically, a single nucleotide, a T residue at position +6 of the 5’ss (Fig 2B), distinguishes therian mammalian sequences from those in other vertebrates. This +6T residue occurs at the third position of a Thr codon, where it has no impact on the amino acid sequence, but it is critical for TRα2 splicing [4]. In platypus the 5’ss +6 position is A rather than T (i.e. AAG/GTGACA in platypus vs. AAG/GTGACT in other mammals where / indicates the 5’ss; Fig 2B).

A previous study of the expression of transfected chimeric opossum-rat minigenes demonstrated that sequences corresponding to the TRα2 5’ss in opossum efficiently support splicing even though opossum does not express TRα2 mRNA [3]. To extend these results, a parallel series of chimeric transcripts was prepared for splicing *in vitro* by replacing exon 9 sequences in the rat minigene pα2ΔBS [32,33] with corresponding sequences from opossum, platypus or chicken. As shown in Fig 2A, a chimeric transcript with exon 9 from opossum underwent splicing *in vitro* as efficiently as the parental rat minigene transcript, a result similar to that obtained in the previous *in vivo* study [3]. However, both the chicken and platypus chimeras were spliced at a cryptic site located 57 nt downstream from the authentic site (position 128 vs. position 185 in exon 9). The sequence of the cryptic site in platypus and chicken (AAG/GTGGAGT) is a good match to the 5’ss consensus sequence (i.e., AAG/GTGAGT) except for inclusion of one additional nucleotide. In contrast, in eutherian and marsupial mammals the corresponding sequence is always AAA/GTCGAGT, with non-consensus nucleotides at positions -1 (A vs. G) and +3 (C vs. G). Thus, platypus is an outlier among mammals in lacking core sequences at both the 5’ and 3’ splice sites.

Based on the observations above, it seems likely that ablation of the downstream cryptic site is important for maintaining TRα2 splicing accuracy. To test this hypothesis, three residues (+6T at the normal 5’ss and -1A and +3C at the cryptic 5’ss) within exon 9 were each replaced with G in the rat transcript to match the sequence found in chicken (Fig 2B). When this transcript (Rat(3G)) was incubated under splicing conditions it underwent similar splicing as seen with the platypus and chicken chimeras (Fig 2A). All bird and reptile sequences currently in GenBank have G residues at positions -1 and +3 at this site, whereas A and C residues are always found in mammals other than platypus. Furthermore, almost all non-therian vertebrates have a nucleotide other than T at the +6 position relative to the TRα2 5'ss. Thus, at each of these positions critical for accurate 5’ss recognition the platypus sequence more closely resembles non-mammalian species than it does therian mammals.

### Conservation of functional elements within Rev-erbα 3’ UTR

The 3’ UTR of Rev-erbα mRNA played an important role in the evolution of TRα2, as it is antisense to the TRα2 3’ss and extends throughout most of the region separating the TRα1 poly(A) site and the Rev-erbα coding sequence (Fig 1B). A compilation of 3’ expressed sequence tags (3’ ESTs) from Rev-erbα in three mammalian species reveals two clusters of polyadenylated 3’ ends (S6 Fig). Approximately two-thirds of these ESTs map close to a site 290 nucleotides from the Rev-erbα stop codon, designated the major poly(A) site (Fig 1B). Most of the other ESTs map to a site 155 nt upstream. The latter site (the minor site; Fig 1B) is located about 14 nt downstream of a well-conserved hexanucleotide, UAUAAA, within the AT-rich region. In contrast to the minor site, the major site lacks an upstream sequence closely resembling the canonical poly(A) signal sequence (PAS) AAUAAA. Instead, a conserved AT-rich region is present immediately upstream (Fig 1B). To investigate requirements for Rev-erbα 3’ end formation 3’ RACE PCR was used to map the 3’ ends of platypus Rev-erbα mRNAs and the effects of mutations on the polyadenylation of rat Rev-erbα minigene.

3’ RACE on total platypus liver RNA yielded a single major product corresponding in size to polyadenylation at the minor poly(A) site of eutherian mammals (S7B Fig, lane 1). As this product proved difficult to sequence directly due to contamination with a non-specific background, two additional rounds of 3’RACE amplification were performed with nested primers (see S7A Fig). The major product from the second round of PCR was successfully sequenced and confirmed polyadenylation near the minor site (S7B Fig, lane 2; S7C Fig). Using primers located downstream of the minor site, additional products were isolated and sequenced. One product corresponded to polyadenylation at the major poly(A) site (S7 Fig, lane 5). Additional 3’RACE products mapped to two closely spaced sites about 240 nt downstream of the major poly(A) site (S7B Fig, lane 6). The latter sites define transcripts that overlap the 3’ end of TRα1 mRNA by 53–60 nt. This result is consistent with two other observations: first, a number of reads from the platypus RNA-Seq data [29] extend across the region separating the 3’ ends of Rev-erbα and TRα1 mRNAs, suggesting transcriptional overlap between the two genes; and second, a few 3’ ESTs from eutherian species also map near these sites within the 3’ end of TRα1 mRNA (for example, GenBank ESTs AA998969 and DN874316). The positions of the 3’ Rev-erbα poly(A) sites in platypus are shown in Fig 1B and S7C Fig.

**Fig 3:**
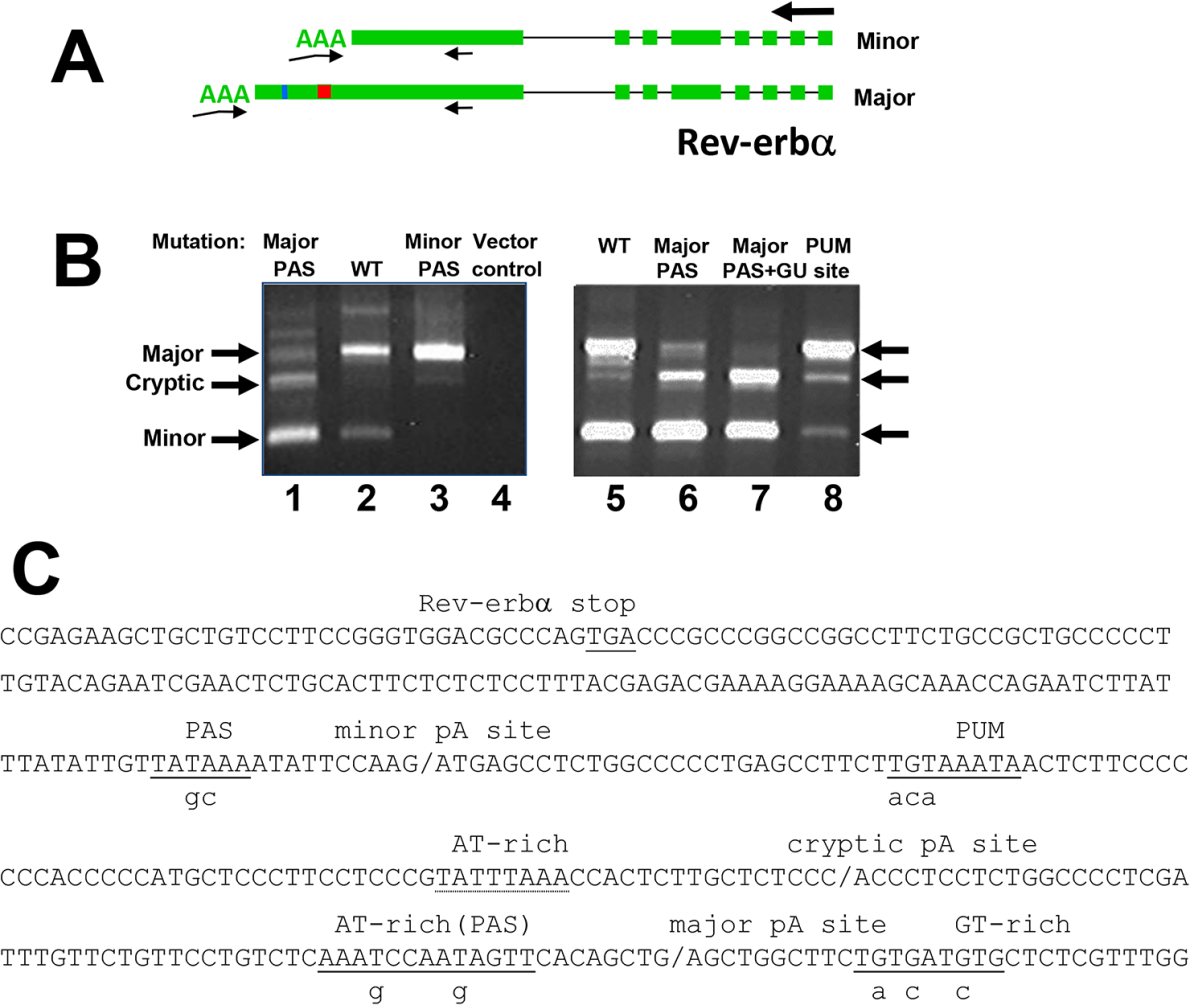
Analysis of conserved sequences required for alternative polyadenylation of Rev-erbα mRNA. (**A)** Schematic showing structure of two Rev-erbα mRNAs alternatively polyadenylated at the minor (top) and major (bottom) sites as indicated in Fig 1A. Red box between the two poly(A) sites represents the position of the PUF protein consensus binding site (PUM) and vertical line the position of the cryptic poly(A) site. Large arrow at right indicates direction of transcription; small arrows indicate primers, with 3’ RACE primers indicated as bent pair of arrows. (**B)** 3’ RACE mapping of alternative poly(A) sites with the rat Rev-erbα minigene. PCR products from 3’RACE of mRNA from cells transfected with Rev-erbα minigenes were analyzed by gel electrophoresis. Lanes 1–4 show effect of mutations on the poly(A) signal sequences upstream of the major and minor poly(A) sites. Lanes 5–8 show effects of additional mutations. Arrows indicate three 3’RACE products corresponding to the major and minor alternatively polyadenylated Rev-erbα mRNAs and RNA polyadenylated at a cryptic site. (**C)** Sequence of 3’ end of Rev-erbα encompassing the poly(A) sites. Conserved elements targeted by mutations are underlined with the substituted nt indicated by small letters below; polyadenylation sites are marked with forward slashes. A non-conserved, AT-rich sequence upstream of the cryptic site is noted with dashed underlining.

The presence of a non-canonical major poly(A) site downstream of a less frequently used canonical poly(A) site is unusual in that non-canonical sites are more commonly situated upstream of a primary canonical site (Tian et al. 2007; Nunes et al. 2010; Tian and Graber 2012; Elkon et al. 2013). To examine the role of the presumptive poly(A) signal elements associated with the major and minor poly(A) sites, a series of mutant rat Rev-erbα minigenes were expressed in transfected cells and assayed via 3’ RACE (Fig 3). A two-nucleotide substitution within the minor site PAS efficiently blocked polyadenylation at the minor site, thus confirming the role of this element in promoting use of the minor poly(A) site (Fig 3B, compare lanes 2 and 3). A similar substitution within the AT-rich element upstream of the major site partially inhibited polyadenylation at the major site and also activated cryptic polyadenylation at an upstream cryptic site (Fig 3B, lanes 1 and 2; Fig 3C). To further characterize requirements for major site polyadenylation, additional substitutions were introduced within the downstream GT-rich element. These substitutions led to a further decrease of polyadenylation at the major site along with further enhancement of cryptic site polyadenylation (Fig 3B, compare lanes 6 and 7).

The presence of multiple independent and conserved poly(A) sites suggests that alternative polyadenylation may provide a mechanism for differential regulation of Rev-erbα expression. A highly conserved 8-nucleotide sequence matching the Pumilio/FBF (PUF) protein consensus binding site located between the major and minor poly(A) sites was investigated, as these proteins regulate mRNA stability and translation [34–36]. Mutation of the PUF protein consensus binding site (PUM) in the rat Rev-erbα minigene (TGTAAATA to acaAAATA; Fig 3C) resulted in a strong increase in usage of the major poly(A) site relative to the minor one (Fig 3B, compare lanes 5 and 8). This result suggests that the wildtype element destabilizes the longer mRNA polyadenylated at the major polyadenylation site. It is possible the PUM site also represses use of the upstream minor site.

### The conserved G30 region in eutherian mammals plays an important role in modulating TRα2 splicing

Several features suggest that the G30 region may play a role in regulating TRα2 splicing. First, it is tightly conserved in diverse eutherian mammals but not in marsupials (S5C Fig), and it is the only region within the overlap between TRα2 and Rev-erbα mRNAs that is not conserved between marsupials and eutherian mammals. Second, it is close to the TRα2 3’ss and antisense to the Rev-erbα 3’UTR and therefore is not constrained by coding requirements for Rev-erbα. Third, it has an unusual sequence composition (63% G, 0% T) and includes several G clusters that are known to play various roles in splice site selection [33,37–42]. Finally, bioinformatic analysis via RESCUE [43] suggests the presence of an exonic splicing enhancer near the center of G30.

**Fig 4:**
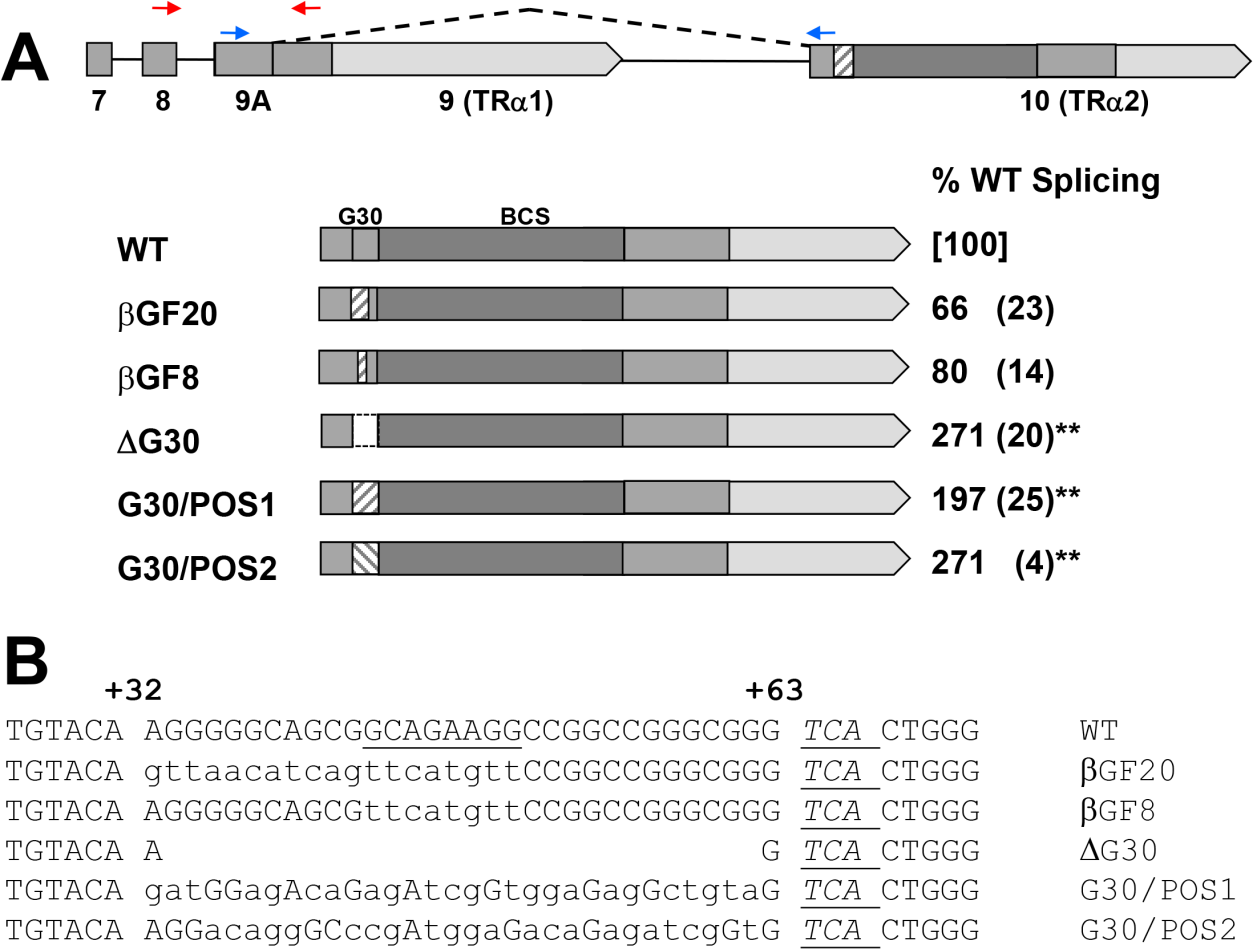
Substitutions and deletions within the G30 region affect TRα2 splicing. (**A)** Schematic representation of exon 10 of TRα2 showing substitutions within the G30 region. Diagram at top shows intron/exon structure of erbAm minigene (exons 7–10). Dark shading within exon 10 indicates bidirectional coding sequence (BCS) where coding sequences for TRα2 and Rev-erbα overlap; medium shading represents other coding sequence; light shading 3’UTR. Small red and blue arrows indicate positions of RT-PCR primers for TRα1 and TRα2 mRNAs, respectively. Lower diagrams indicate structure of exon 10 for wildtype (WT) and five mutations. Substitution of homologous sequences from either Rev-erbβ gene (βGF) or opossum Rev-erbα gene (POS) are indicated as hatched boxes; open box indicates deletion of G30 (ΔG30). TRα2 splicing, measured by real-time RT-PCR, is given at right as % of wildtype splicing (standard deviation), N = 3; wildtype splicing = 28% (SD 8.2%). Asterisks indicate the significance of the change in expression of TRα2 in mutant compared to WT as determined by Student’s t test (* p< 0.05, ** p < 0.01). (**B)** Sequences of the G30 region for wildtype and mutant constructs shown in panel A. Positions +33 to +63, as determined from 5’ end of exon 10, correspond to G30. Flanking sequences are shown, including BsrGI site (TGTACA) used in construction and sequence antisense to Rev-erbα stop codon (*TCA*). Underlined WT sequence corresponds to a predicted exonic splicing enhancer. Substituted nt are shown in small letters.

Initial experiments involved the substitution or deletion of substantial portions of the G30 region in the ErbAm minigene [4], followed by expression in transfected cells (Fig 4) and measurement of TRα2 splicing by real-time RT-PCR. Substitution of 8 or 20 bp with corresponding sequences derived from the homologous Rev-erbβ gene (GF8, GF20 in Fig 4) resulted in small to moderate decreases in TRα2 splicing consistent with the disruption of a splicing enhancer. However, a large deletion of 30 nt (ΔG30) and two extensive substitutions (POS-1, POS-2) within the G30 region resulted in dramatic 2- to 3-fold *increases* in TRα2 splicing (Fig 4).

**Fig 5:**
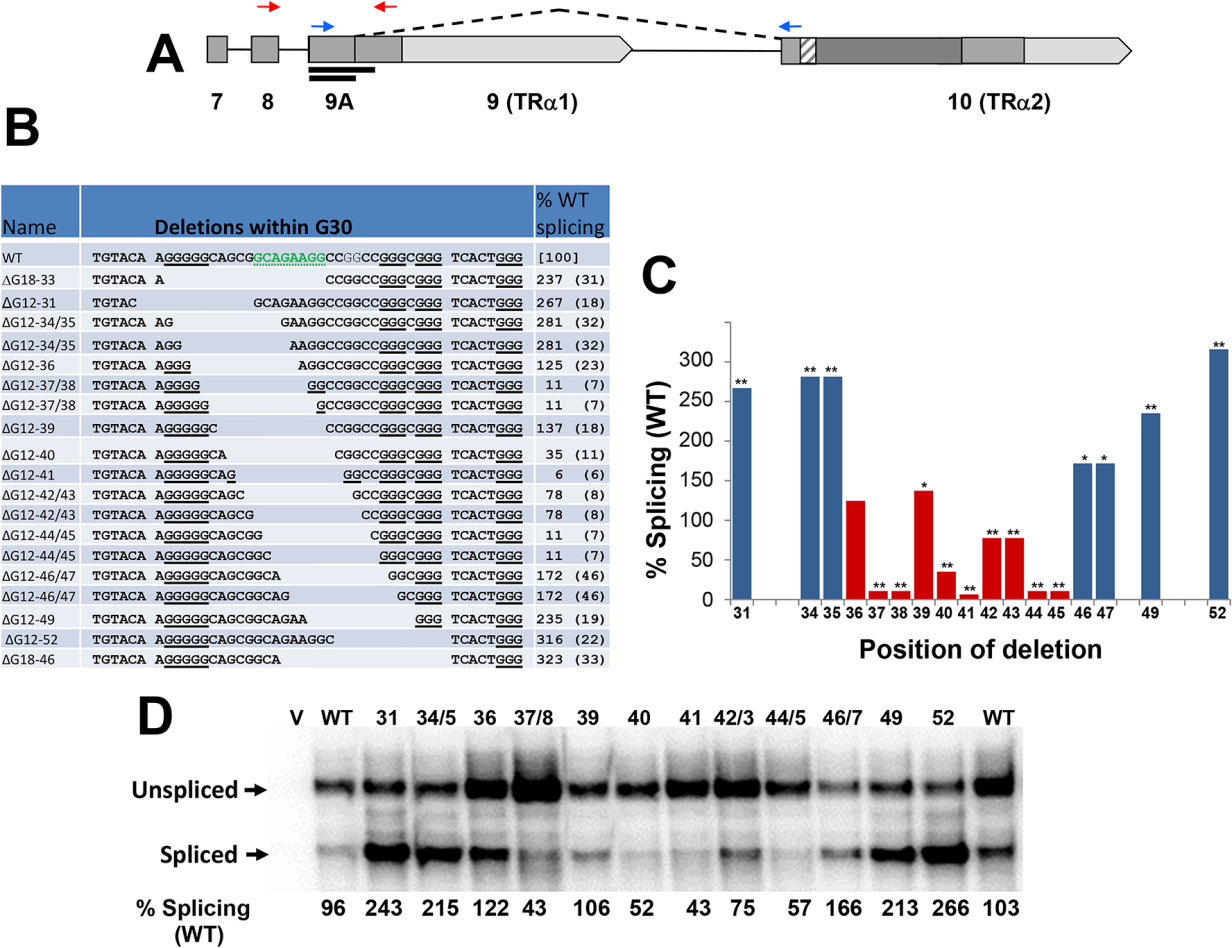
Role of G-clusters in G30 probed by deletion scanning mutations. **(A)** Diagram of the structure of the ErbAm minigene as in Fig 4A. The longer line under exon 9 represents the riboprobe used for RNase protection assays in panel D, the shorter line RNase protected probe annealed to spliced TRα2 mRNA. (**B)** Sequences of closely spaced 12 and 18 nucleotide deletions (ΔG12, ΔG18) are indicated within the G30 region. Deletions are named according to the position of their 5’ ends in exon 10. Clusters of three or more G residues are underlined. The possible 8-nt splicing enhancer sequence is highlighted in the wildtype sequence. The positions of some deletions are ambiguous, and these are shown twice to emphasize stepwise positioning of the deletions. TRα2 splicing determined by real-time RT-PCR is shown at right as % of wildtype splicing with standard deviation given in parentheses (N = 3–6); WT = 25% TRα2 splicing (SD 6.2%). (**C)** Bar graph summarizing results from ΔG12 deletions in panel A. Ambiguous positions are indicated with two identical bars; blue bars indicate deletions which disrupt at least one G3 cluster. One or two asterisks indicate the significance of the change in expression of TRα2 in mutant compared to WT (p < 0.05 or p <0.01, respectively). (**D)** Autoradiogram showing results from RNase protection assays carried out in parallel using a probe complementary to the TRα2 5’ss in exon 9. Minigene deletions are labeled similarly to panel B with ambiguous positions indicated with forward slash.

To systematically examine the stimulatory effects of the deletions in the G30 region, a series of 12 and 18 bp deletions was tested (ΔG12 and ΔG18 mutations; Fig 5A and 5B). These smaller deletions included a series of nine 12 bp deletions spaced one nucleotide apart. These deletions displayed a range of effects, varying from strong enhancement to strong inhibition of TRα2 splicing. Deletions at either end of the G30 region (for example, ΔG12-31, ΔG12-33/34 and ΔG12-52) were uniformly associated with a 2- to 3-fold increase in splicing activity, while deletions within the center of the G30 (ΔG12-37/38, ΔG12-41, ΔG12-44/45) were strongly inhibitory (<80% wildtype; Fig 5C). To confirm these results based on real-time RT-PCR, RNA from transfected cells was also analyzed by RNase protection assays (RPAs) using a labeled probe complementary to the TRα2 5’ss (Fig 5D). These assays directly measure the activity of the 5’ss for TRα2. Results of the RPAs displayed trends very similar to the real-time PCR assays. Both assays gave similar levels of TRα2 splicing for wildtype splicing (25+/-6.2% TRα2 splicing vs. 28+/-1% splicing for qRT-PCR vs RPA assays, respectively); however, RPA assays of minigenes expressing the lowest levels of TRα2 showed 43–57% wildtype splicing vs 6–35% wildtype measured by real-time PCR. The lower level of inhibition and enhancement measured by the RPA assays reflects the lower dynamic range of these assays, which are complicated by background splicing within the host cell [3].

Examination of the sequences of each of the ΔG12 mutations suggests that deletions that disrupt clusters of three or more G residues (Fig 5A, bold underlining) result in strong increases in splicing, whereas those that extend the G-clusters (or otherwise decrease the spacing between them) strongly inhibit splicing. Deletions at the 5’ end that disrupted a run of five G residues in G30 all strongly enhanced splicing, with the exception of ΔG12-36, which retains a run of three consecutive G residues. In contrast, deletions that strongly inhibited splicing added to the density of G clusters within this region by either extending the G_5_ run (ΔG12-37/38), adding another (ΔG12-41), or reducing the spacing between existing ones (ΔG12-44/45). The correlation of TRα2 splicing levels with absence or presence of densely spaced clusters of G residues within the G30 region is consistent with the presence of an inhibitory structure, possibly a G-quadruplex.

**Fig 6:**
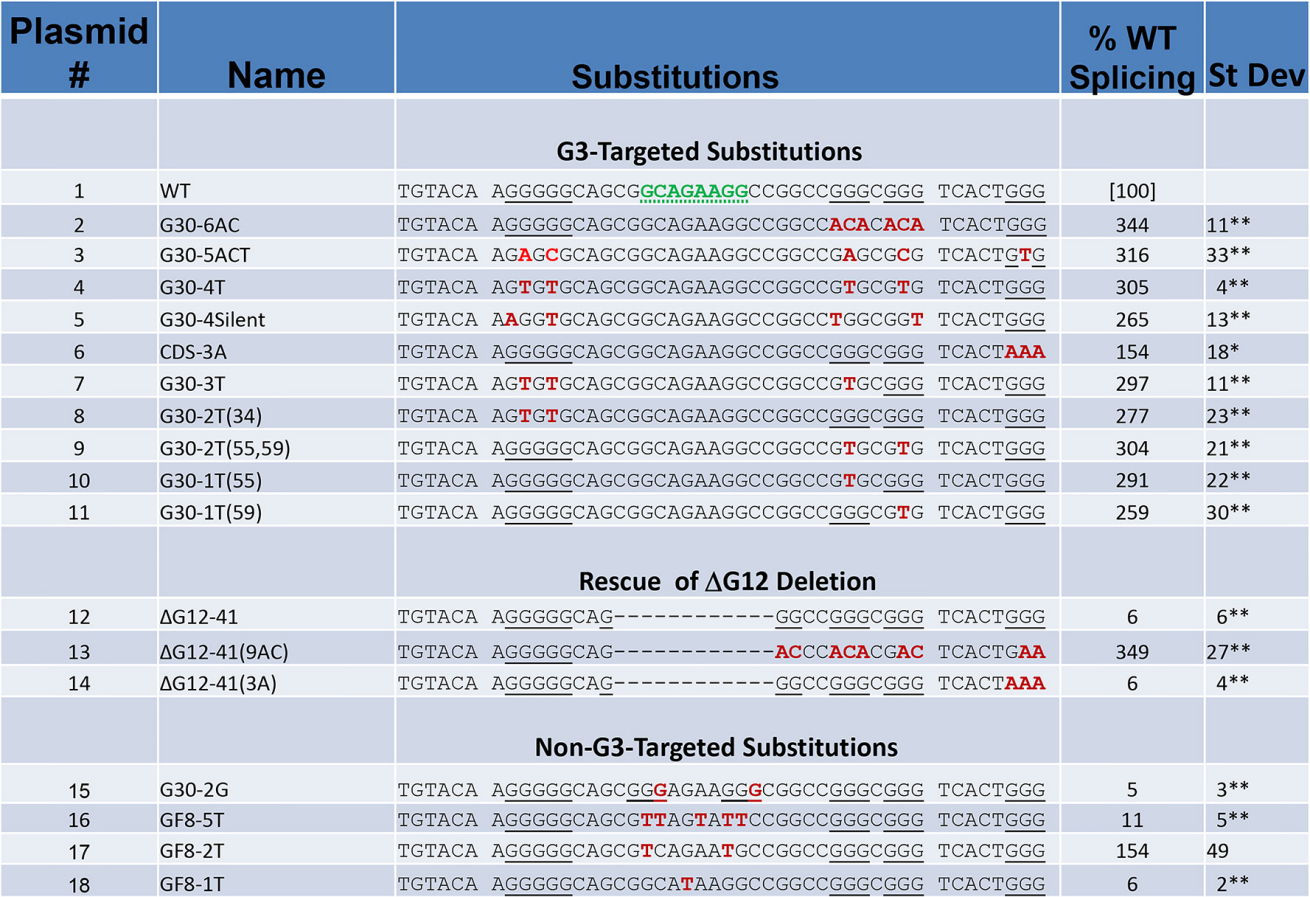
Substitutions within G30 support a critical role for G clusters in regulating TRα2 splicing. Substitutions within the G30 region of minigene ErbAm are shown with results of real-time RT-PCR assays as in Fig 5B (N = 3–6).

Further experiments were carried out by introducing targeted substitutions in G30 and an adjacent G_3_ cluster. As shown at the top of Fig 6 (plasmids 1–11), nucleotide substitutions that disrupt multiple G clusters in exon 10 uniformly resulted in a 2- to 3-fold increase in splicing. In one construct, G30-4Silent (plasmid 5), four substitutions were introduced into G30 in such a way that only silent mutations were introduced into the TRα2 coding sequence. This mutation, like others that disrupted G clusters in G30, led to a substantial increase in TRα2 splicing. Furthermore, disruption of the G_3_ clusters present in the ΔG12-41 construct (ΔG12-41(9AC)) rescued splicing inhibited by this deletion (Fig 6, compare plasmids 12–13). In contrast, introduction of two C to G substitutions adjacent to G_2_ dinucleotides produced two additional G_3_ clusters (G30-2G, plasmid 15) and resulted in a strong decrease in splicing. These results all demonstrate that increasing the number or density of G clusters within G30 strongly inhibits TRα2 splicing, but disrupting the clusters present in the wildtype G30 greatly enhances TRα2 splicing.

To rigorously examine the requirement for multiple G clusters in the full-length G30, substitutions were made in each of three G clusters within G30, which were disrupted by one or two T residues (either singly or in pairs). In each of these five mutations (Fig 6: plasmids 7–11) TRα2 splicing was strongly enhanced, suggesting that each cluster individually is important for maintaining wildtype levels of splicing. These results are consistent with a model in which all three G clusters in G30 contribute to a single functional structure, such as a G-quadruplex [44].

These results also suggest possible roles for sequences within the G30 element. Since all strongly inhibitory mutations also disrupt the putative 8-nucleotide enhancer sequence highlighted in the wildtype sequence (Figs 4–6), constructs with one to five T-substitutions at positions between the first two G clusters were tested. Two of these strongly inhibited splicing (Fig 6, plasmids 16 and 18: GF8-1T and GF8-5T), but another (plasmid 17, GF8-2T) appeared to enhance splicing. Thus, while strong enhancement of splicing is associated with disruption of one of the three G clusters, it is likely that other sequences within G30 sequences are important for promoting splicing of TRα2. In this context the relatively modest splicing inhibition observed with GF-20 may result from a combination of opposing effects, a balance between disrupting elements that positively and negatively regulate TRα2 splicing. Thus, G30 may play a dual role in regulating the balance between TRα1 and TRα2 expression.

Since stable G-quadruplex structures are often found in sequences with four closely spaced clusters of G residues, we considered the possible role of a fourth closely spaced G_3_ cluster located close to G30 and antisense to the Rev-erbα coding sequence. However, disruption of this G cluster, in the context of the wildtype sequence (Fig 6; plasmid 6), had a relatively modest effect on splicing. The same mutation within a ΔG12 deletion (Fig 6; compare plasmids 12 and 14) had no apparent effect. These results suggest that the G4 structure associated with the wildtype G30 may be comprised of G residues within the G30 region itself. Such a structure might plausibly be formed by recruiting G residues from non-adjacent sites in the sequence into a G-quadruplex as seen in other G4 structures [45,46].

### The effect of mutations in G30 on *in vitro* splicing of TRα2 pre-mRNA

The strong enhancement of TRα2 splicing associated with multiple mutations that disrupt G clusters within G30 suggests that these mutations all disrupt a cooperative structure. For example, multiple closely spaced G clusters within the pre-mRNA may form a stable, multilayered G-quadruplex that can affect protein-RNA interactions important for mediating the effects of splicing regulatory elements [44,47,48]. Alternatively, formation of a G-quadruplex on the non-template strand of DNA might indirectly alter splicing through its effect on transcription or TRα1 polyadenylation. In fact, G-rich sequences generally, and G-quadruplexes in particular, promote R-loop formation in the wake of the transcribing RNA polymerase. [30,49,50].

**Fig 7:**
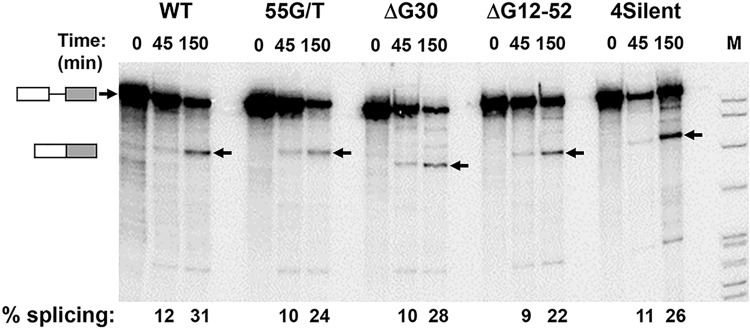
Mutations in the G clusters of G30 have no effect on splicing of TRα2 pre-mRNA *in vitro*. Four mutations that greatly enhance splicing of rat TRα2 minigenes when expressed *in vivo* were incorporated into the pα2- ΔBS minigene. Rat TRα2 pre-mRNAs with or without four mutations described in Figs 4–6 were incubated under splicing conditions as shown in Fig 2. Small arrows indicate unspliced RNAs at top and spliced products at bottom. Percent splicing is calculated from phosphoimager scans after correcting for nucleotide composition of the spliced and unspliced RNAs.

To address the possible link between transcription and the involvement of the G30 element in regulating the balance between TRα1 and TRα2 processing, four different mutations that enhanced splicing in transfection assays were tested in an *in vitro* splicing system. These mutations include two deletions (ΔG30, Fig 4; and ΔG12-52, Fig 5), a single-base point mutant (G30-1T(55), Fig 6, plasmid 10) and four substitutions that disrupted all three G-clusters within G30 (G30-4Silent, Fig 6, plasmid 5). Each of these mutations was incorporated into the rat α2- ΔBS minigene, the same construct used to analyze 5’ss requirements (Fig 2A). As shown in Fig 7, transcripts with each of these mutations were spliced *in vitro* at the same rate as the wildtype control. These results suggest that the splicing enhancement observed in *in vivo* studies with the same G30 mutants requires processing linked to active transcription of the transfected minigene.

## Discussion

In this study phylogenetic analysis is used to identify cis-acting elements important for regulation of TRα and Rev-erbα, two closely linked genes that play important roles in developmental and metabolic regulation. In particular, we have characterized a short exonic G-rich element (designated G30) that is tightly conserved among eutherian mammals but absent in marsupials and platypus. The dramatic effects of different mutations within G30 suggest that it plays a critical role in regulating the balance between TRα1 and TRα2 mRNA. These results highlight the potential importance of post-transcriptional events, in addition to transcriptional-level controls [9], in regulating the expression of these nuclear receptor proteins. Our results also provide insight into the evolution of the alternatively spliced eutherian TRα2 isoform that may further aid in understanding the regulation of these important nuclear receptor protiens.

### Evolution of TRα2 mRNA

Formation of novel alternatively spliced exons provides a widespread mechanism for evolution of species-specific functions within metazoan animals [51,52]. Comparative analysis suggests several features of the TRα/Rev-erbα locus that were specifically required for the evolution of TRα2 mRNA. Most important of these are, first, the convergent transcription of the two genes, apparently an ancient feature conserved in many non-mammalian vertebrates and within the paralogous TRβ/Rev-erbβ locus and, second, the proximity of the TRα1 and Rev-erbα mRNAs. The proximity of the two genes is greatest in mammals and birds, where the coding regions of TRα1 and Rev-erbα are separated by only 2.8–3.8 kb, in comparison with other vertebrates such as lizard, frog and turtle, where the separation is much greater (9–15 kb).

The increased proximity of TRα and Rev-erbα in mammals (and birds) relative to that in other vertebrates implies an increased probability of transcriptional overlap between the genes since termination of mRNA transcription is largely a stochastic process which typically occurs some distance downstream of the polyadenylation site. Such readthrough of TRα transcription across the Rev-erbα gene would have provided the substrate for the evolution of TRα2 mRNA in mammals. However, an increase in overlapping transcription would also increase the possibility of transcriptional interference or collision of RNA polymerases that can impact the expression and the integrity of the genes, a situation that may select for more efficient termination [53,54]. Consistent with this possibility, the close spacing of TRα and Rev-erbα genes in birds and mammals is associated with a large number of clusters of G and C residues that are skewed in their distribution relative to the 3’ ends of the genes, a feature particularly evident in eutherian mammals. Similar skewing of G- and C-rich composition has been noted at the 3’ ends of other convergently transcribed genes, further supporting the suggestion that these sequences are associated with transcriptional termination [31].

Transcriptional termination is also closely linked mechanistically to mRNA polyadenylation [55]. We have identified a distal site in platypus (S7 Fig) that is antisense to the 3’ end of TRα1 mRNA, in addition to the major and minor Rev-erbα poly(A) sites. The presence of this downstream poly(A) site in platypus is clear evidence for transcriptional overlap between the two genes even in the absence of TRα2. This overlap falls within the highly conserved region at the 3’ end of TRα1 mRNA (Fig 1B). The back-to-back poly(A) signal sequences are associated with a large AT-rich palindrome that is among the most highly conserved features of this locus (S7C Fig), suggesting that it might have played a role in the early evolution of transcriptional overlap between the TRα and Rev-erbα genes.

Comparison of the platypus TRα/Rev-erbα locus with the corresponding sequences of other mammals and non-mammalian vertebrates suggests a specific pathway for the evolution of the TRα2/Rev-erbα antisense overlap. In some respects the platypus genes more closely resemble those of birds and reptiles, most notably in the absence of core 5’ss and 3’ss elements directly involved in TRα2 splicing. On the other hand, platypus, marsupials and eutherian mammals all share elements in the 3’ UTR that are likely important for regulation of Rev-erbα expression but are largely missing in other vertebrates. The coding capacity of the nucleotide sequence of the final exon of Rev-erbα mRNA provides another useful metric for comparing sequences from which TRα2 evolved. The Rev-erbα amino acid sequence in exon 8 is nearly identical in marsupials and eutherian mammals and only slightly different in platypus (S2 Fig), consistent with strong conservation of the Rev-erbα LBD. However, virtual translation of the sequence antisense to the exon 8 coding sequence of platypus (and birds) yields an amino acid sequence that is more similar to the eutherian TRα2 sequence than to corresponding sequences in marsupials (S4 Fig). Strikingly, stop codons that interrupt the various marsupial sequences on the strand antisense to the Rev-erbα coding sequences are absent in platypus (S4A Fig). In summary, while platypus and marsupials both lack TRα2, the many differences between these two mammalian lineages suggest that they have quite different evolutionary histories.

This comparative analysis supports a scenario previously described as “TRα2 lost” (see Fig 6 in [3]), in which TRα2 alternative splicing evolved prior to the marsupial-eutherian divergence. This model provides a plausible explanation for TRα2 evolution in mammals and can be elaborated based on observed properties of newly evolved, species-specific exons, such as those associated with Alu element exonization in primates [51,56]. Detailed studies show that newly evolved exons are most often associated with weak, inefficient splice sites [57,58]. This presumably reflects the fact that novel splicing pathways are likely to be deleterious and thus are more readily tolerated if they are infrequently utilized. However, novel exons also provide a basis for further evolutionary experimentation, in which efficiency of splicing and its regulation can be modified in a stepwise fashion.

These considerations suggest that TRα2 mRNA originated with infrequent alternative splicing of a read-through transcript in a common ancestor of marsupials and eutherian mammals, sometime after the divergence of monotremes and therian mammals. In the lineage leading to modern marsupials, TRα2 mRNA may have failed to develop as a fully functional product. In the absence of positive selection pressures, poorly expressed version(s) of TRα2 were eventually lost in marsupial ancestors, but with retention of certain vestigial features, including the splicing-competent 5’ss and the 3’ss polypyrimidine tract. In the eutherian lineage the sequence of exon antisense to Rev-erbα evolved to provide a functional TRα2 protein whose regulated expression accommodated the competing demands for alternative processing of two abundantly expressed mRNAs as well as overlapping antisense transcription of Rev-erbα. The multiple constraints on TRα2 sequence and expression are reflected in the exceptional conservation of sequence evident both upstream and downstream of the TRα2 3’ss, while the corresponding sequences in marsupial were free to diverge further from those of the common mammalian and therian ancestors [3,32].

### Role of the G30 element in regulating expression of the TRα/Rev-erbα locus

The requirements for the regulated expression of novel exons in the model discussed above suggest that the G30 element may have evolved as part of a broad array of changes necessary to establish well-regulated TRα2 expression in eutherian mammals. Thus G-rich sequences present in platypus, birds and turtle at the same position as G30 in eutherian mammals may play a role in transcriptional termination downstream of the TRα1 poly(A) site [30,31]. It is possible that G30 may have retained a related function, but one modified by additional requirements for maintaining a balance between the alternatively processed TRα1 and TRα2 mRNAs. The diverse effects of substitution and deletion mutations within the G30 element on TRα2 splicing suggest that the G30 region may have a dual function, acting both to enhance and repress TRα2 splicing. For example, the formation of higher order structure within G30 may inhibit splicing by inducing transcriptional pausing downstream of the TRα2 3’ss, simultaneously stimulating TRα1 polyadenylation upstream as illustrated in the model in S8 Fig. On the other hand, destabilization of such higher order structure in G30 may expose an embedded exonic splicing enhancer element, making it accessible to proteins that promote splicing. Thus, G30 may function as a molecular switch whose activity is determined by protein binding to nascent RNA and by transcriptional activity across the site.

Although the tendency of guanosine nucleotides to form planar G-quartets and multilayer quadruplexes is well documented, the biological functions of these structures in the context of DNA and RNA metabolism are poorly understood [44,59]. G-quadruplexes have been shown to disrupt DNA replication and transcription through multiple mechanisms, including R-loop formation as shown in S8 Fig [50,60,61]. Several studies suggest that G-quadruplexes are involved in regulating splicing, 3’ end formation and transcriptional termination [62–64,65]. G-quadruplexes form structures in both DNA and RNA whose stability is sensitive to the binding of specific monovalent cations and other ligands [44,60,66]. Both enzymatic and non-enzymatic proteins associated with RNA or DNA may destabilize G-quadruplexes [44,48,60,67,68]. Among proteins that bind preferentially to G-rich sequences, binding of hnRNP F/H proteins, which are implicated in mRNA splicing and polyadenylation, has been shown to compete with formation of G-quadruplex structure [44,48]. Thus, the higher order structure of G-rich sequence, such as that in G30, may affect splicing both through its effects on transcription and its interactions with proteins involved in spliceosome activity.

In conclusion, these studies raise a number of interesting questions concerning the mechanisms associated with alternative splicing of a bidirectionally transcribed locus. Mutational analysis of the G30 element suggests that higher order structure of RNA or DNA may provide a mechanism for linking transcription and alternative processing at the TRα1 and TRα2 mRNAs. Other work also suggests that antisense transcription impacts gene expression at many levels, affecting both local and global gene regulation [53,54,69–76]. A better understanding of antisense regulation is important given the ubiquity of antisense transcription in the genomes of diverse organisms [69–74].

## Materials and Methods

### Ethics statement

Platypus samples were collected and held under The Australian National University Animal Experimentation Ethics Committee proposal numbers R.CG.11.06 and R.CG.14.08, as previously described [77].

### DNA and RNA Sequencing

Short unassigned contigs from the platypus genome project that closely matched portions of mammalian TRα or Rev-erbα mRNAs were identified and used as a starting point for PCR amplification of genomic sequences spanning exons 4–9 of TRα and exons 6–8 of Rev-erbα (S1 Fig). Platypus spleen DNA was amplified with OneTaq polymerase (New England Bio Labs). Two longer PCR products were cloned prior to sequencing; shorter ones were sequenced directly. The resulting sequences were assembled into a continuous 7021 bp sequence extending from the beginning of exon 4 of TRα to exon 6 of Rev-erbα. This sequence included 4352 bp from four previously determined contigs (NW_001728154.1, NW_001765690.1, NW_001785919.1, NW_001641166.1) and 5120 bp of new sequence obtained from amplifying. The overlap between new and old sequence, representing 35% of total length, was > 99% identical. Most of the discrepancies between the old and new sequence appeared to represent sequencing errors located near the ends of the previously deposited sequences. For example, a 121 bp overlap between NW_001785919.1 and NW_001641166.1 across the exon 7/intron 7 junction (S1 Fig) included numerous mismatches. These mismatches, and others, were resolved in favor of newly determined sequence and confirmed by multiple rounds of sequencing.

Genomic sequencing was augmented by assembly of the coding sequence of platypus TRα1 and Rev-erbα mRNAs and adjacent non-coding regions obtained by assembling Illumina reads generated in a previous study and deposited in the Gene Expression Omnibus (primary accession GSE30352; accession GSM752569, run SRR306725, and accession GSM752570 run SRR306727; [29]). *De novo* assembly was carried out with the Oases Package [78] using k values between 21 and 73. A single consensus transfrag covers the entire TRα1 coding sequence (exons 2–9), and two non-overlapping transfrags cover all but 25 nt of the Rev-erbα coding sequence (exons 1–8). The missing nucleotides, which mapped within a highly repetitive region in exon 5 of Rev-erbα, were obtained by sequencing a 500 bp PCR product. The assembled mRNA sequences were nearly identical to overlapping portions of the genomic consensus sequence and were further checked by aligning the assembled mRNA sequences with individual reads. Two positions were identified in Rev-erbα mRNA displaying >30% sequence variation that may represent possible polymorphisms (C690G, T704C). Genomic and TRα1 and Rev-erbα mRNA sequences from platypus have been deposited in GenBank under accession numbers KR028100, KR020832 and KR020833, respectively.

### Sequence analysis

Sequence analysis was carried out using tools from Vector NTI (Life Technologies), the EMBL-EBI Web Site (http://www.ebi.ac.uk/Tools/msa/clustalo/) and the SIB ExPASy Web Site (http://www.expasy.org/). The following Rev-erbα mRNA sequences were obtained from GenBank: Norway rat NM_001113422.1, human NM_021724.4, gray short-tailed opossum (M. domestica) XM_001370259, green anole lizard XM_003222449.2. The following TRα1 sequences were used: rat NM_001017960.1, human NM_199334.2, chicken NM_205313.1, green anole lizard XM_008113350.1, gray short-tailed opossum NM_001197206.1. Genomic sequences include human NC_000017.11, rat NC_005109.4, gray short-tailed opossum NC_008802.1.

### Plasmid construction

Minigene plasmids were constructed by inserting PCR fragments containing the desired mutations into previously described plasmid constructs [3,4,8,32,33]. Transfection assays were carried out with plasmids derived from pCMV-erbAm [4], which contains an intact 9.1 kb segment from rat TRα/Rev-erbα locus, extending from exon 7 of TRα1 to exon 2 of Rev-erbα expressed from the hCMV promoter. To facilitate multiple constructs with mutations located in exon 10 of TRα2, pCMV-erbAm was modified by individually removing via end-filling two EcoRI sites: one located within a non-conserved region of the TRα1 3’ UTR 950 bp upstream of the poly(A) site, and the other within the vector, pRcCMV. The resulting construct, pErbAR, was used to construct a series of mutants by substituting appropriate sequences between the unique BsrGI and EcoRI sites in exon 10 of TRα2. Rev-erbα polyadenylation was investigated using plasmid constructs derived from pCMV-Revα, a Rev-erbα minigene which expresses exons 2–8 of Rev-erbα from the same genomic fragment present in pErbAm. pCMV-Revα was constructed by reversing the orientation of the insert in pErbAm so that exons 2–8 of Rev-erbα were positioned downstream of the hCMV promoter between the NotI and ApaI sites of the vector. Mutations in pCMV-Revα were constructed by inserting mutagenized recombinant PCR products in pCMV-Revα cut with AgeI and BsrGI.

Two series of plasmids were constructed from pα2-ΔBS, a previously described TRα2 minigene that is used for preparing labeled transcripts for splicing *in vitro* [8,33]. In the first series of chimeric transcripts the rat sequence present in exon 8/9 cDNA was replaced with the corresponding sequence from chicken, opossum and platypus by inserting PCR-generated fragments between HindIII and Bsu36I sites (Fig 2). Another series of pα2 minigenes was prepared with mutations in G30 by inserting appropriate PCR products between the BsrGI and EcoRI sites (Fig 7). Details of these and other constructions are described in S1 Table. The sequences of all PCR-generated mutations in plasmids were confirmed by sequencing the full length of the inserts and are available upon request.

### Transfection assays, *in vitro* splicing and RNA analysis

Minigene plasmids were transfected into HEK-293 cells (ATCC CRL-1573) for 48 h, and total RNA was isolated by phenol/chloroform extraction and DNase treated as previously described [4]. TRα2 splicing was measured by real-time RT-PCR and as described previously [3] using primers that were targeted to exons 8 and 9 of TRα1 mRNA and exons 9A and 10 of TRα2 mRNA (S1 Table). All realtime RT-PCR assays were carried out with triple replicas and at least three independent transfection experiments. % splicing was calculated from realtime RT-PCR data assuming 100% amplification efficiency as described previously [3]. The significance of changes in splicing relative to the wildtype control was evaluated by the Student’s two sample unpooled t test. Parallel assays were carried out with primers to host cell beta-actin to control for quality of the RNA and transfection efficiency. RNase protection assays were carried out using a ^32^P-labeled riboprobe to exon 9 as previously described [4] with 1 μg of RNA per assay. TRα2 splicing was calculated after correcting for the base composition of the protected fragments.

*In vitro* splicing was carried out by incubating gel-purified, ^32^P-labeled pre-mRNAs in HeLa cell nuclear extracts as previously described [33]. Direct sequencing of RT-PCR products was used to identify spliced RNAs obtained following transfection assays and *in vitro* splicing. 3’ RACE analysis was carried out using SuperScript II reverse transcriptase (Life Technologies) followed by amplification with GoTaq polymerase (Promega). RACE primers were obtained from Integrated DNA Technology and primers derived from the 3’ sequence of Rev-erbα mRNA (S1 Table). RACE products were gel-purified and either sequenced directly or sequenced after cloning into pGEM-T (Promega).

## Supporting Information

S1 Fig Strategy for sequencing the TRα/Rev-erbα locus in the platypus genome. Arrows at top indicate direction of TRα1 and Rev-erbα mRNA transcription with exons and intron structure shown below with numbered boxes for the exons. Four contigs identified by homology to human genes are labeled as segments from A-D (solid lines). A and A’ represent two sequences within same contig (GenBank NW_ 001765690.1). Overlapping PCR products (ab, bc, ca’ and a’d, dotted lines) obtained from amplifying platypus DNA were sequenced on both strands and used to assemble a genomic sequence extending from exon 4 of TRα1 mRNA to exon 6 of Rev-erbα. The tables below summarize information about contigs used in sequencing the platypus TRα/Rev-erbα locus (left) and the identity of primers used in PCR-based sequencing (right).
(PDF)

S2 Fig Comparison of the nucleotide sequence of platypus Rev-erbα ligand binding domain with sequences from mammals, birds and reptiles. **(A)** Alignment of representative Rev-erbα amino acid sequences. Boxed region corresponds to core LBD structure shown in panel B; arrow indicates exon7/exon 8 boundary. **(B)** Alignment of nucleotide sequences coding for the Rev-erbα exon 8 coding sequence (BCS). At top are the amino acid sequences of the corresponding sequences for human Rev-erbα and TRα2 (italics, antisense strand). Residues differing in platypus Rev-erbα or rat are underlined. (**C)** Crosstables giving the percent amino acid identity for Rev-erbα from different vertebrates. (**D)** Crosstable giving the percent nucleotide identity of Rev-erbα mRNA sequences in the LBD and BCS. GenBank accessions for Rev-erbα protein sequences are NP_068370.1 (human), NP_001106893.1 (rat), XP_001370296.1 (gray short-tailed opossum), ADG08189.1 (long-nosed potoroo), XP_005232747.1 (Peregrine falcon), XP_005531577.1 (Tibetan ground-tit), XP_005294178.1 (Western painted turtle), XP_003222497.1 (green anole lizard), and NP_001093675.1 (frog, *X*. *tropicalis*). Rev-erbα nucleotide sequences from GenBank are rat NM_001113422.1 (rat), NM_021724.4 (human), XM_005294121 (western painted turtle), XM_005531520 (Tibetan ground tit), XM_005232690.1 (Peregrine falcon), HM149328.1 long-nosed potoroo, XM_001370259 (gray short-tailed opossum, M.dom.), HM149332 (Virginia opossum, D.vir.), XM_003222449.2 (green anole lizard) and KR020833 (platypus).
(PDF)

S3 Fig Comparison of platypus TRα1 sequences with orthologous proteins and mRNAs from other vertebrates. **(A)** Alignment of TRα1 amino acid sequences from representative vertebrates (mammals, bird and reptile). Boxed region shows sequences adjacent to the TRα2 5’ss (arrow) shown in panel B. **(B)** Conservation in diverse vertebrates of the nucleotide sequences adjacent to the TRα2 5’ss in eutherian mammals. Boxes highlight the 5’ss consensus sequences and arrows indicate position of splice sites as in Fig 2B. The consensus amino acid sequence is shown at bottom with a single Met/Thr replacement in platypus indicated. (**C)** Crosstables giving percent identity for pairwise comparisons of aligned amino acid sequences for TRα1. The following files were used: TRα1 sequences are NP_955366 (human), NP_001017960 (rat), NP_001184135.1 (gray short-tailed opossum), ADG08190.1 (long-nosed potoroo), NP_990644.1 (chicken), XP_005531484.1 (Tibetan ground-tit), XP_005294177.1 (Western painted turtle), XP_003222498.1 (green anole lizard), and NP_001039261.1 (frog, *X*. *tropicalis*). TRα1 mRNA sequences are NM_001017960 (rat), NM_199334.2 (human), XM_005861866.1 (Brandt’s bat), NM_001286862.1 (dog), XM_004282755.1 (killer whale), XM_005220734.2 (cow), XM_004378062.1 (manatee), XM_006889407.1 (elephant shrew), NM_001197206.1 (gray short-tailed opossum, M.dom.), HM149329.1 (potoroo), XM_003768259.1 (Tasmanian devil), KR020832 (platypus), NM_205313.1 (chicken), XM_005531427.1 (Tibetan ground tit), XM_008636216.1 (crow), XM_005294120.1 (western painted turtle), XM_008113350.1 (green anole lizard), XM_007420682.1 (Burnese python), AB204861.1 (gecko), XM_006270785.1 (American alligator), NM_001045796.1 (western clawed frog), AAO47435.1 (axolotl), XM_001920978 (zebrafish), XM_006638232.1 (spotted gar), XM_006006382.1 (coelacanth), XM_011603682.1 (Fugu rubripes), XM_010866384.1 (northern pike).
(PDF)

S4 Fig Comparison of the amino acid sequence from exon 10 of TRα2, which is antisense to the coding sequence of Rev-erbα, and the virtual translations of sequences antisense to the Rev-erbα coding sequence in exon 8 of platypus, marsupials, birds and reptiles. (**A)** Alignment of 67 amino acids from the bidirectional coding sequence. X indicates a stop codon. Related species (reptiles/birds, marsupials and eutherian mammals) are grouped together and a consensus sequence is given for each group. Amino acids differing from the consensus are highlighted. Residues in the platypus sequence that differ from the eutherian consensus are also highlighted (**B)** Crosstable based on alignment in panel A giving the number of amino acid *differences* between the 67 codons corresponding to the BCS of TRα2. The following files were used for comparison of TRα2-related sequences: NP_003241.2 (human), NP_112396.2 (rat), NC_006591.3 (dog), XP_004378116.1 (Florida manatee), HM149331.1 (long nosed-potoroo), BK007078.1 (tammar wallaby), HM149332.1 (Virginia opossum), XM_001370259 (gray short-tailed opossum), NW_004929918 (Peregrine falcon), NW_005087648 (Tibetan ground-tit), NW_003338727.1 (green anole lizard), and NW_004848538.1 (western painted turtle).
(PDF)

S5 Fig Distribution of G and C clusters in region between the TRα1 poly(A) site and the G30-homologous regions preceding the Rev-erbα stop codon. **(A)** Clusters of three or more G or C residues are highlighted in red or green, respectively. The signal elements (PAS) for polyadenylation of TRα1 and Rev-erbα are boxed; arrow indicates position of the TRα2 splice site in rat and human. Sequences are referenced in S2–S4 Figs. (**B)** Comparison of the G-rich regions adjacent and antisense to the Rev-erbα stop codon (italicized) and the corresponding regions in marsupials, platypus, birds and turtle. Sequences are aligned at left to highlight similarities among diverse vertebrates. The 6 nt flanking regions at top left overlap the final 6 nt shown in panel A. **(C)** Conservation of sequence within diverse eutherian mammals. Sequences flanking the G30 region are indicated; the (antisense) stop codon for Rev-erbα is italicized and underlined. Two variant positions are indicated in the G30 sequence in pika and cow. Sequence files include pika (XM_004591164.1), dog (XM_003435250), ferret (XM_004764619.1), panda (XM_002924928), cow (NM_001046329.1), killer whale (XM_004282753.1), horse (XR_131463), star-nosed mole (XM_004684230.1), armadillo (NW_004465229), hedgehog tenrec (XR_193663.1), manatee (XM_004378060.1), bald eagle (XM_010585709.1), collared flycatcher (XM_005059802.1), and wallaby (BK007078) in addition to other Rev-erbα mRNAs in S2 Fig.
(PDF)

S6 Fig Distribution of Rev-erbα poly(A) sites in GenBank collection of 3’ expressed sequence tags (ESTs) from human (blue bars), mouse (red bars) and rat (yellow bars). ESTs were sorted according to position of poly(A) sites. These formed two clusters representing the minor upstream poly(A) site (upper chart) and the major downstream site (lower chart). Positions are given relative to the rat Rev-erbα coding sequence (cds) and to the conserved PAS sites for each poly(A) site. Note different scales for each chart.
(PDF)

S7 Fig 3’RACE mapping of poly(A) sites in platypus Rev-erbα mRNA. (**A)** Diagram showing relative positions of nested primers (A-D) as in Fig 3A. (**B)** Gel electrophoresis of 3’RACE products of platypus liver RNA following reverse transcription and multiple rounds of 3’RACE PCR amplification. Lane 1: first round 3’RACE with upstream primer A; lanes 2–5 second round 3’ RACE PCR using product shown in lane 1 with primers B, A, C and D, respectively. Products from lanes 2 and 5 were sequenced and correspond to polyadenylation at the minor and major poly(A) sites, respectively. Lane 6 shows the third round PCR product obtained by amplifying primer C 3’RACE product (from lane 4) with primer D. This product when sequenced identified a downstream polyadenylation site. Its anomalous size reflects mispriming within the C_n_ region. Products from isolated bands in lanes 2 and 6 were sequenced both directly and after cloning. Band from lane 5 was sequenced after cloning. Asterisks (*) indicate PCR reactions subjected to second (lane 1) and third (lane 4) rounds of amplification. Arrows indicate bands sequenced. (**C)** Sequence of Rev-erbα 3’ UTR in platypus. The positions of the four poly(A) sites are indicated (/). The upstream PAS and AT-rich sequences are underlined, with the TRα1 PAS on the opposite strand indicate by the dotted line.
(PDF)

S8 Fig Model for linkage of splicing and transcription as mediated by the G30 element. G-clusters within the G30 region (red box) and within the intron (red circles) may promote RNA polymerase pausing (light blue ellipses) and R-loop formation in a transcription-dependent manner as represented schematically by parallel dotted orange (RNA) and blue (DNA) lines within intron. Pausing near the G30 element, near the 3’ ss may inhibit TRα2 splicing and promotes TRα1 polyadenylation as indicated by curved arrow. Large blue arrows represent 3’ exons and poly(A) sites for TRα1 and TRα2 mRNAs. The effect of G-clusters on pausing and R-loop formation may involve G-quadruplex structure on either the displaced strand of DNA (irregular blue line) or the RNA transcript.
(PDF)

S1 Table DNA primer sequences used for DNA sequencing, PCR, 3’ RACE and plasmid construction. (XLS)
