# Supplementary material for: Evolution of the Antisense Overlap between Genes for Thyroid Hormone Receptor and Rev-erbα and Characterization of an Exonic G-Rich Element That Regulates Splicing of TRα2 mRNA
Source: PLoS One. 2015 Sep 14;10(9):e0137893. doi: 10.1371/journal.pone.0137893 (PMC4569393; doi:10.1371/journal.pone.0137893)
Supplement: S1 Fig — Arrows at top indicate direction of TRα1 and Rev-erbα mRNA transcription with exons and intron structure shown below with numbered boxes for the exons. Four contigs identified by homology to human genes are labeled as segments from A-D (solid lines). A and A’ represent two sequences within same contig (GenBank NW_ 001765690.1). Overlapping PCR products (ab, bc, ca’ and a’d, dotted lines) obtained from amplifying platypus DNA were sequenced on both strands and used to assemble a genomic sequence extending from exon 4 of TRα1 mRNA to exon 6 of Rev-erbα. The tables below summarize information about contigs used in sequencing the platypus TRα/Rev-erbα locus (left) and the identity of primers used in PCR-based sequencing (right). (PDF) [file pone.0137893.s001.pdf]

A

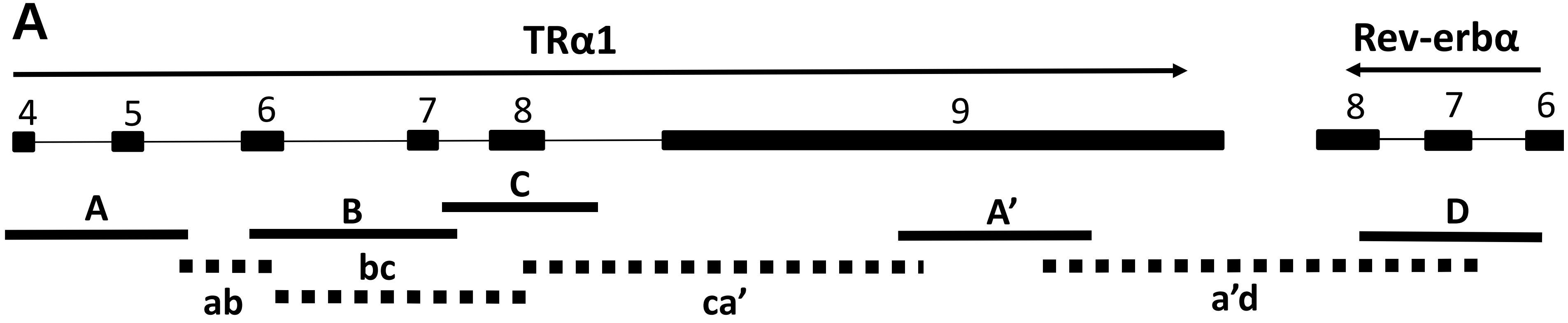

B

TRα1/Rev-erbα contigs

GenBank Accession #	Segment	Length	Genome Position	Exon Match	Exon Position	Exon Length
NW_001765690.1*	**A**	840	1-840	TRα1 exon 4	1-101	101
				TRα1 exon 5	458-605	148
	**A'**	885	4741-5634	TRα1 exon 9	4750-5634	2578
NW_001641166.1	**B**	969	1111-2069	TRa1 exon 6	1111-1269	206
				TRa1 exon 7	1826-1972	147
NW_001755919.1	**C**	815	1949-2763	TRa1 exon 7	1949-1972	147
				TRa1 exon 8	2198-2457	259
NW_001728154.1	**D**	836	6186-7021	Rev-erba exon 8	6276-5985	292
				Rev-erba exon 7	6476-6686	211
				Rev-erba exon 6	6949-7021	186

- includes 486 bp of intron 3 (not shown)

C

New Sequence

Sequence	Primers	Start	End	Length
**ab**	22-81F	794	1279	486
	22-82R			
**bc**	22-83R	1260	2418	1159
	22-84F			
**ca'**	22-63	2382	4864	2483
	22-67			
**a'd**	22-38	5420	6661	1242
	22-50			
