# Supplementary material for: Evolution of the Antisense Overlap between Genes for Thyroid Hormone Receptor and Rev-erbα and Characterization of an Exonic G-Rich Element That Regulates Splicing of TRα2 mRNA
Source: PLoS One. 2015 Sep 14;10(9):e0137893. doi: 10.1371/journal.pone.0137893 (PMC4569393; doi:10.1371/journal.pone.0137893)
Supplement: S2 Fig — (A) Alignment of representative Rev-erbα amino acid sequences. Boxed region corresponds to core LBD structure shown in panel B; arrow indicates exon7/exon 8 boundary. (B) Alignment of nucleotide sequences coding for the Rev-erbα exon 8 coding sequence (BCS). At top are the amino acid sequences of the corresponding sequences for human Rev-erbα and TRα2 (italics, antisense strand). Residues differing in platypus Rev-erbα or rat are underlined. (C) Crosstables giving the percent amino acid identity for Rev-erbα from different vertebrates. (D) Crosstable giving the percent nucleotide identity of Rev-erbα mRNA sequences in the LBD and BCS. GenBank accessions for Rev-erbα protein sequences are NP_068370.1 (human), NP_001106893.1 (rat), XP_001370296.1 (gray short-tailed opossum), ADG08189.1 (long-nosed potoroo), XP_005232747.1 (Peregrine falcon), XP_005531577.1 (Tibetan ground-tit), XP_005294178.1 (Western painted turtle), XP_003222497.1 (green anole lizard), and NP_001093675.1 (frog, X. tropicalis). Rev-erbα nucleotide sequences from GenBank are rat NM_001113422.1 (rat), NM_021724.4 (human), XM_005294121 (western painted turtle), XM_005531520 (Tibetan ground tit), XM_005232690.1 (Peregrine falcon), HM149328.1 long-nosed potoroo, XM_001370259 (gray short-tailed opossum, M.dom.), HM149332 (Virginia opossum, D.vir.), XM_003222449.2 (green anole lizard) and KR020833 (platypus). (PDF) [file pone.0137893.s002.pdf]

A

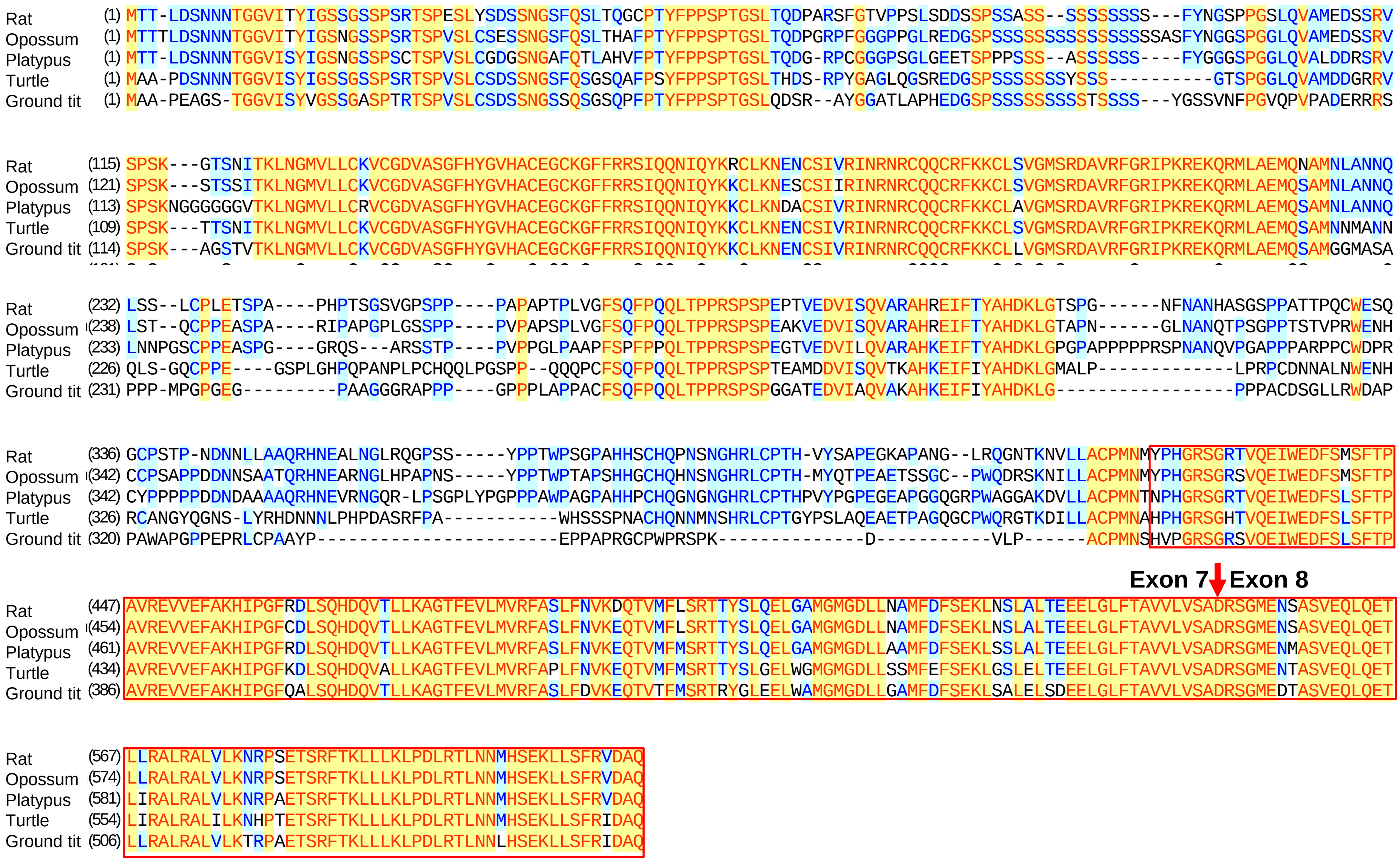
Rat (1) MTT-LDSNNNTGGVITYIGSSGSSPSRTSPESLYSDSSNGSFQSLTQGCPTYFPPSPTGSLTQDPARSFGTVPPSLSDDSSPSSASS--SSSSSSSS---FYNGSPPGSLQVAMEDSSRV
Opossum (1) MTTTLDSNNNTGGVITYIGSNGSSPSRTSPVSLCSESSNGSFQSLTHAFPTYFPPSPTGSLTQDPGRPFGGGPPGLREDGSPSSSSSSSSSSSSSSSASFYNGGSPGGLQVAMEDSSRV
Platypus (1) MTT-LDSNNNTGGVISYIGSNGSSPSCTSPVSLCGDGSNGAFQTLAHVFPTYFPPSPTGSLTQDG-RPCGGGPSGLGEETSPPPSSS--ASSSSSS----FYGGGSPGGLQVALDDRSRV
Turtle (1) MAA-PDSNNNTGGVISYIGSSGSSPSRTSPVSLCSDSSNGSFQSGSQAFPSYFPPSPTGSLTHDS-RPYGAGLQGSREDGSPSSSSSSYSSS----------GTSPGGLQVAMDDGRRV
Ground tit (1) MAA-PEAGS-TGGVISYVGSSGASPTRTSPVSLCSDSSNGSSQSGSQPFPTYFPPSPTGSLQDSR--AYGGATLAPHEDGSPSSSSSSSSTSSSS---YGSSVNFPGVQPVPADERRRS
Rat (115) SPSK---GTSNITKLNGMVLLCKVCGDVASGFHYGVHACEGCKGFFRRSIQQNIQYKRCLKNENCSIVRINRNRCQQCRFKKCLSVGMSRDAVRFGRIPKREKQRMLAEMQNAMNLANNQ
Opossum (121) SPSK---STSSITKLNGMVLLCKVCGDVASGFHYGVHACEGCKGFFRRSIQQNIQYKKCLKNESCSIIRINRNRCQQCRFKKCLSVGMSRDAVRFGRIPKREKQRMLAEMQSAMNLANNQ
Platypus (113) SPSKNGGGGGGVTKLNGMVLLCRVCGDVASGFHYGVHACEGCKGFFRRSIQQNIQYKKCLKNDACSIVRINRNRCQQCRFKKCLAVGMSRDAVRFGRIPKREKQRMLAEMQSAMNLANNQ
Turtle (109) SPSK---TTSNITKLNGMVLLCKVCGDVASGFHYGVHACEGCKGFFRRSIQQNIQYKKCLKNENCSIVRINRNRCQQCRFKKCLSVGMSRDAVRFGRIPKREKQRMLAEMQSAMNNMANN
Ground tit (114) SPSK---AGSTVTKLNGMVLLCKVCGDVASGFHYGVHACEGCKGFFRRSIQQNIQYKKCLKNENCSIVRINRNRCQQCRFKKCLLVGMSRDAVRFGRIPKREKQRMLAEMQSAMGGMASA
Rat (232) LSS--LCPLETSPA----PHPTSGSVGPSPP----PAPAPTPLVGFSQFPQQLTPPRSPSPEPTVEDVISQVARAHREIFTYAHDKLGTSPG------NFNANHASGSPPATTPQCWESQ
Opossum (238) LST--QCPPEASPA----RIPAPGPLGSSPP----PVPAPSPLVGFSQFPQQLTPPRSPSPEAKVEDVISQVARAHREIFTYAHDKLGTAPN------GLNANQTPSGPPTSTVPRWENH
Platypus (233) LNNPGSCPPEASPG----GRQS---ARSSTP----PVPPGLPAAPFSPFPPQLTPPRSPSPEGTVEDVILQVARAHKEIFTYAHDKLGPGPAPPPPPRSPNANQVPGAPPPARPPCWDPR
Turtle (226) QLS-GQCPPE----GSPLGHPQPANPLPCHQQLPGSPP--QQQPCFSQFPQQLTPPRSPSPTEAMDDVISQVTKAHKEIFIYAHDKLGMALP------------LPRPCDNNALNWENH
Ground tit (231) PPP-MPGPGEG---------PAAGGGRAPPP----GPPPLAPPACFSQFPQQLTPPRSPSPGGATEDVIAQVAKAHKEIFIYAHDKLG----------------PPPACDSGLLRWDAP
Rat (336) GCPSTP-NDNNLLAAQRHNEALNGLRQGPSS-----YPPTWPSGPAHHSCHQPNSNGHRLCPTH-VYSAPEGKAPANG--LRQGNTKNVLLACPMNMYPHGRSGRTVQEIWEDFSMSFTP
Opossum (342) CCPSAPPDDNNSAATQRHNEARNGLHPAPNS-----YPPTWPTAPSHHGCHQHNSNGHRLCPTH-MYQTPEAETSSGC--PWQDRSKNILLACPMNMYPHGRSGRSVQEIWEDFSMSFTP
Platypus (342) CYPPPPPDDNDAAAAQRHNEVRNGQR-LPSGPLYPGPPPAWPAGPAHHPCHQGNGNGHRLCPTHPVYPGPEGEAPGGQGRPWAGGAKDVLLACPMNTNPHGRSGRTVQEIWEDFSLSFTP
Turtle (326) RCANGYQGNS-LYRHDNNNLPHPDASRFPA----------WHSSSPNACHQNNMNSHRLCPTGYPSLAQEAETPAGQGCPWQRGTKDILLACPMNAHPHGRSGHTVQEIWEDFSLSFTP
Ground tit (320) PAWAPGPPEPRLCPAAYP----------------------EPPAPRGCPWPRSPK-------------D-----------VLP------ACPMNSHVPGRSGRSVQEIWEDFSLSFTP
Exon 7
Exon 8
Rat (447) AVREVVEFAKHIPGFRDLSQHDQVTLLKAGTFEVLMVRFASLFNVKDQTVMFLSRTTYSLQELGAMGMGDLLNAMFDFSEKLNSLALTEEELGLFTAVVLVSADRSGMENSASVEQLQET
Opossum (454) AVREVVEFAKHIPGFCDLSQHDQVTLLKAGTFEVLMVRFASLFNVKEQTVMFLSRTTYSLQELGAMGMGDLLNAMFDFSEKLNSLALTEEELGLFTAVVLVSADRSGMENSASVEQLQET
Platypus (461) AVREVVEFAKHIPGFRDLSQHDQVTLLKAGTFEVLMVRFASLFNVKEQTVMFMSRTTYSLQELGAMGMGDLLAAMFDFSEKLSSLALTEEELGLFTAVVLVSADRSGMENMASVEQLQET
Turtle (434) AVREVVEFAKHIPGFKDLSQHDQVALLKAGTFEVLMVRFAPLFNVKEQTVMFMSRTTYSLGELWGMGMGDLLSSMFEFSEKLGSLELTEEELGLFTAVVLVSADRSGMENTASVEQLQET
Ground tit (386) AVREVVEFAKHIPGFQALSQHDQVTLLKAGTFEVLMVRFASLFDVKEQTVTFMSRTRYGLEELWAMGMGDLLGAMFDFSEKLSALELSDEELGLFTAVVLVSADRSGMEDTASVEQLQET
Rat (567) LLRALRALVLKNRPSETSRFTKLLLKLPDLRTLNNMHSEKLLSFRVDAQ
Opossum (574) LLRALRALVLKNRPSETSRFTKLLLKLPDLRTLNNMHSEKLLSFRVDAQ
Platypus (581) LIRALRALVLKNRPAETSRFTKLLLKLPDLRTLNNMHSEKLLSFRVDAQ
Turtle (554) LIRALRALILKNHPTETSRFTKLLLKLPDLRTLNNMHSEKLLSFRIDAQ
Ground tit (506) LLRALRALVLKTRPAETSRFTKLLLKLPDLRTLNNLHSEKLLSFRIDAQ

B

```
           ValAlaArgAlaHisLeuIleGlySerArgHisLeuLeuGluLeuLeuArgGlnGlnProSerLysProSerGlnHisGlnLeuValProGlyGlnLeuSer hTRα2
           AspArgSerGlyMetGluAsnSerAlaSerValGluGlnLeuGlnGluThrLeuLeuArgAlaLeuArgAlaLeuValLeuLysAsnArgProSerGluThr hRev-erbα
Human       ACCGCTCGGGCATGGAGAATTCCGCTTCGGTGGAGCAGCTCCAGGAGACGCTGCTGCGGGCTCTTCGGGCTCTGGTGCTGAAGAACCGGCCCTTGGAGACT
Rat         ACCGCTCGGGAATGGAGAATTCCGCTTCGGTGGAGCAGCTCCAGGAGACGCTGCTGCGGGCTCTTCGGGCTCTGGTGCTGAAGAACCGGCCCTCGGAGACT
Opossum     ATCGATCAGGTATGGAGAACTCGGCCTCTGTAGAACAGCTGCAGGAGACGCTGCTCCGGGCTCTTCGGGCCCTGGTCCTGAAGAATCGGCCCTCAGAGACC
Potoroo     ACCGATCAGGTATGGAGAACTCAGCCTCTGTGGAACAGTTGCAGGAGACACTGCTCCGAGCACTTCGGGCACTGGTCCTAAAGAACAGGCCCTCAGAGACA
Platypus    ACCGCTCGGGCATGGAGAACATGGCATCGGTGGAGCAGCTGCAGGAGACGCTGATCCGGGCGCTGCGGGCCCTGGTGCTGAAGAACCGCCCGGCCGAGACG
Ground tit  ACCGCTCGGGCATGGAGGACACGGCATCAGTGGAGCAGCTGCAGGAGACGCTGCTGCGGGCCCTGCGCGCCCTCGTGCTGAAGACGCGCCCGGCTGAGACC
Falcon      ACCGCTCGGGCATGGAGGACACGGCATCGGTGGAGCAGCTGCAGGAGACGCTGATCCGCGCCCTGCGCGCCCTCGTGCTGAAGACGCACCCGGCGGAGACA
Lizard      ATCGCTCTGGCATTGAGAACCAGGCATCAGTGGAGCAGCTGCAGGAGACACTAATCCGGGCTCTTCGGGCCCTCATCCTCAAGAATCACCCCCAGGAGACT
Turtle      ACCGCTCCGGCATGGAGAACACGGCGTCAGTGGAGCAGCTGCAGGAGACCCTGATCCGTGCCCTGCGCGCTGATCCTCAAGAACCACCCCACAGAGACC

            GlyAlaGluGlyLeuGlnGlnGluLeuGlnArgValGlnProGlyGlnValValHisMetGlyLeuLeuGlnGlnGlyGluProHisValGlyLeuSer    hTRα2
            SerArgPheThrLysLeuLeuLeuLysLeuProAspLeuArgThrLeuAsnAsnMetHisSerGluLysLeuLeuSerPheArgValAspAlaGln(stop) hRev-erbα
Human       TCCCGCTTCACCAAGCTGCTGCTCAAGCTGCCGGACCTGCGGACCCTGAACAACATGCATTCCGAGAAGCTGCTGTCCTTCCGGGTGGACGCCCAGTGA
Rat         TCCCGCTTCACCAAACTGCTGCTCAAGCTGCCGGACCTGCGGACCCTGAACAACATGCATTCCGAGAAGCTGCTGTCCTTCCGGGTGGACGCCCAGTGA
Opossum     TCCCGCTTCACCAAGCTGCTGCTTAAGCTGCCTGACCTGCGCACCCTCAACAACATGCATTCCGAGAAGTTGCTGTCCTTCCGAGTAGATGCCCAGTGA
Potoroo     TCCCGCTTCACCAAGCTACTGCTTAAGCTGCCTGACCTCCGTACCCTCAACAACATGCATTCCGAGAAGCTGCTGTCCTTCCGAGTAGATGGCCAGTGA
Platypus    TCCCGCTTCACCAAGCTGCTGCTCAAGCTGCCCGATCTGCGCACGCTGAACAACATGCATTCCGAGAAGCTCCTGTCCTTCCGCGTGGACGCCCAGTGA
Ground tit  TCGCGCTTCACCAAACTGCTGCTGAAGCTGCCGGACCTGCGCACTCTCAACAACCTCCACTCCGAGAAGCTGCTCTCCTTCCGCATCGACGCCCAGTAG
Falcon      TCGCGCTTCACCAAACTGCTGCTGAAGCTGCCGGACCTGCGCACCCTCAACAACCTCCACTCCGAGAAGCTGCTCTCCTTCCGCATCGACGCCCAGTAG
Lizard      TCCCGCTTCACCAAACTGCTGCTGAAGCTTCCTGACCTGCGCACCCTCAACAACATGCACTCTGAGAAACTGCTCTCCTTCCGCATTGATGCGCAGTAG
Turtle      TCGCGCTTCACCAAGCTGCTGCTGAAGCTGCCCGACCTGCGCACGCTGAACAACATGCATTCGGAGAAGCTGCTGTCCTTCCGCATCGACGCCCAGTAG
```

C

Rev-erbα (% amino acid identity)

	Platypus	Human	Rat	Opossum	Potoroo	Falcon	Tit	Turtle	Lizard
Human	78.9								
Rat	78.4	95.0							
Opossum(M.dom)	80.5	86.0	84.9						
Potoroo	80.4	86.0	84.7	97.6					
Falcon	68.8	68.4	67.4	68.7	68.3				
Ground tit	72.2	72.0	71.5	72.2	71.6	90.3			
Turtle	73.2	75.0	74.2	77.0	76.0	71.3	73.7		
Lizard	71.5	72.5	71.7	74.8	73.9	70.4	72.3	78.4	
Frog	69.0	69.5	68.5	69.5	69.3	65.1	66.9	70.5	68.7

Rev-erbα LBD (% amino acid identity)

	Platypus	Human	Rat	Opossum	Potoroo	Falcon	Tit	Turtle	Lizard
Human	95.6								
Rat	95.6	98.9							
Opossum(M.dom)	95.6	97.8	98.9						
Potoroo	95.1	97.3	98.4	99.5					
Falcon	89.1	86.3	85.8	86.3	85.8				
Ground tit	89.6	87.4	87.4	88.0	87.4	94.5			
Turtle	91.3	89.6	89.1	89.6	89.1	90.7	86.9		
Lizard	88.5	86.3	86.3	86.9	86.3	90.7	88.0	92.9	
Frog	80.9	80.3	79.8	80.3	79.8	77.6	77.1	79.8	77.6

D

Rev-erbα LBD (% Nucleotide Identity)

	Platypus	Human	Rat	Opossum	Potoroo	Falcon	Tit	Turtle
Human	86.9							
Rat	85.7	94.3						
Opossum (M.dom)	82.6	86.0	86.4					
Potoroo	80.8	84.1	83.4	92.4				
Falcon	87.1	82.6	80.7	78.6	77.4			
Ground tit	87.1	81.9	80.5	78.6	76.5	92.9		
Turtle	87.2	83.4	81.2	79.1	78.6	86.4	84.5	
Lizard	78.9	77.4	77.0	78.9	76.3	80.8	78.6	81.7

Rev-erbα Exon 8 CDS (% Nucleotide Identity)

	Platypus	Human	Rat	M.dom	D.vir	Potoroo	Wallaby	Falcon	Tit	Turtle
Human	88.5									
Rat	88.0	98.5								
Opossum (M.dom)	85.0	87.0	87.0							
Opossum (D.vir)	85.0	86.5	86.5	96.5						
Potoroo	81.5	84.5	84.5	91.5	91.5					
Wallaby	82.0	85.0	85.0	92.0	92.0	98.0				
Falcon	86.5	82.0	82.5	80.0	79.5	77.0	77.0			
Ground tit	86.0	82.5	83.0	81.0	80.5	76.5	77.0	96.0		
Turtle	87.0	82.5	82.0	82.5	84.5	80.5	81.0	87.0	86.5	
Lizard	80.5	79.5	79.5	82.0	82.0	77.5	78.5	84.0	82.5	84.0
