# Supplementary material for: Evolution of the Antisense Overlap between Genes for Thyroid Hormone Receptor and Rev-erbα and Characterization of an Exonic G-Rich Element That Regulates Splicing of TRα2 mRNA
Source: PLoS One. 2015 Sep 14;10(9):e0137893. doi: 10.1371/journal.pone.0137893 (PMC4569393; doi:10.1371/journal.pone.0137893)
Supplement: S3 Fig — (A) Alignment of TRα1 amino acid sequences from representative vertebrates (mammals, bird and reptile). Boxed region shows sequences adjacent to the TRα2 5’ss (arrow) shown in panel B. (B) Conservation in diverse vertebrates of the nucleotide sequences adjacent to the TRα2 5’ss in eutherian mammals. Boxes highlight the 5’ss consensus sequences and arrows indicate position of splice sites as in Fig 2B. The consensus amino acid sequence is shown at bottom with a single Met/Thr replacement in platypus indicated. (C) Crosstables giving percent identity for pairwise comparisons of aligned amino acid sequences for TRα1. The following files were used: TRα1 sequences are NP_955366 (human), NP_001017960 (rat), NP_001184135.1 (gray short-tailed opossum), ADG08190.1 (long-nosed potoroo), NP_990644.1 (chicken), XP_005531484.1 (Tibetan ground-tit), XP_005294177.1 (Western painted turtle), XP_003222498.1 (green anole lizard), and NP_001039261.1 (frog, X. tropicalis). TRα1 mRNA sequences are NM_001017960 (rat), NM_199334.2 (human), XM_005861866.1 (Brandt’s bat), NM_001286862.1 (dog), XM_004282755.1 (killer whale), XM_005220734.2 (cow), XM_004378062.1 (manatee), XM_006889407.1 (elephant shrew), NM_001197206.1 (gray short-tailed opossum, M.dom.), HM149329.1 (potoroo), XM_003768259.1 (Tasmanian devil), KR020832 (platypus), NM_205313.1 (chicken), XM_005531427.1 (Tibetan ground tit), XM_008636216.1 (crow), XM_005294120.1 (western painted turtle), XM_008113350.1 (green anole lizard), XM_007420682.1 (Burnese python), AB204861.1 (gecko), XM_006270785.1 (American alligator), NM_001045796.1 (western clawed frog), AAO47435.1 (axolotl), XM_001920978 (zebrafish), XM_006638232.1 (spotted gar), XM_006006382.1 (coelacanth), XM_011603682.1 (Fugu rubripes), XM_010866384.1 (northern pike). (PDF) [file pone.0137893.s003.pdf]

A

```
Rat        (1) MEQKPSKVECGSDPEENSARSPDGKRKRKNGQCPLKSSMSGYIPSYLDKDEQCVVCGDKATGYHYRCITC
Opossum    (1) MEQKPSKLDCGSDPEEN--RSLDGKRKRKTSQCSLKTSMSGYIPSYLDKDEQCVVCGDKATGYHYRCITC
Platypus   (1) MEQKPNTLDCGVDPEEN--RSPDGKRKRKSSQCSLKSSMSGYIPSYLDKDEQCVVCGDKATGYHYRCITC
Chicken    (1) MEQKPSTLDPLSEPEDT--RWLDGKRKRKSSQCLVKSSMSGYIPSYLDKDEQCVVCGDKATGYHYRCITC
Turtle     (1) MEQKPSTLDCLSEPEET--RWLDGKRKRKSSQCSVKSSMSGYIPSYLDKDEQCVVCGDKATGYHYRCITC

Rat       (71) EGCKGFFRRTIQKNLHPTYSCKYDSCCVIDKITRNQCQLCRFKKCIAVGMAMDLVLDDSKRVAKRKLIEQ
Opossum   (69) EGCKGFFRRTIQKNLHPTYSCKYDGCCIIDKITRNQCQLCRFKKCISVGMAMDLVLDDSKRVAKRKLIEE
Platypus  (69) EGCKGFFRRTIQKNLHPTYSCKYDSCCVIDKITRNQCQLCRFKKCIGVGMAMDLVLDDSKRVAKRKLIEE
Chicken   (69) EGCKGFFRRTIQKNLHPTYSCKYDGCCVIDKITRNQCQLCRFKKCISVGMAMDLVLDDSKRVAKRKLIEE
Turtle    (69) EGCKGFFRRTIQKNLHPTYSCKYDGSCVIDKITRNQCQLCRFKKCIAVGMAMDLVLDDSKRVAKRKLIEE

Rat      (141) NRERRRKEEMIRSLQQRPEPTPEEWDLIHVATEAHRSTNAQGSHWKQRRKFLPDDIGQSPIVSMPDGDKV
Opossum  (139) NRERRRKEEMIRSLQQRPEPSPEEWDLIHLVTEAHRSTNAQGSHWKQKRKFLPEDIGQSPMASMPDGDKV
Platypus (139) NRERRRKEEMIKSLQQRPEPTDGEWELIQLVTEAHRSTNAQGSHWKQKRKFLPEDIGQSPIASMPDGDKV
Chicken  (139) NRERRRKEEMIKSLQHRPSPSAEEWELIHVVTEAHRSTNAQGSHWKQKRKFLPEDIGQSPMASMPDGDKV
Turtle   (139) NRERRRKEEMIKTLQHRPEPSAEEWELIHVVTEAHRSTNAQGSHWKQKRKFLPEDIGQSPMASMPDGDKV

Rat      (211) DLEAFSEFTKIITPAITRVVDFAKKLPMFSELPCEDQIILLKGCCMEIMSLRAAVRYDPESDTLTLSGEM
Opossum  (209) DLEAFSEFTKIITPAITRVVDFAKKLPMFSELPCEDQIILLKGCCMEIMSLRAAVRYDPESETLTLSGEM
Platypus (209) DLEAFSEFTKIITPAITRVVDFAKKLPMFSELPCEDQIILLKGCCMEIMSLRAAVRYDPDSETLTLSGEM
Chicken  (209) DLEAFSEFTKIITPAITRVVDFAKKLPMFSELPCEDQIILLKGCCMEIMSLRAAVRYDPESETLTLSGEM
Turtle   (209) DLEAFSEFTKIITPAITRVVDFAKKLPMFSELPCEDQIILLKGCCMEIMSLRAAVRYDPESETLTLSGEM

Rat      (281) AVKREQLKNGGLGVVSDAIFELGKSLSAFNLDDTEVALLQAVLLMSTDRSGLLCVDKIEKSQEAYLLAFE
Opossum  (279) AVKREQLKNGGLGVVSDAIFDLGKSLSAFNLDDTEVALLQAVLLMSSDRSGLLCVDKIEKCQEAYLLAFE
Platypus (279) AVKREQLKNGGLGVVSDAIFDLGKSLSAFNLDDTEVALLQAVLLMSSDRSGLLCVDKIEKCQEAYLLAFE
Chicken  (279) AVKREQLKNGGLGVVSDAIFDLGKSLSAFNLDDTEVALLQAVLLMSSDRTGLICVDKIEKCQETYLLAFE
Turtle   (279) AVKREQLKNGGLGVVSDAIFDLGKSLSAFNLDDTEVALLQAVLLMSSDRTGLLRVEKIEKCQETYLLAFE

                                  TRα2 5'ss
Rat      (351) HYVNHRKHNIPHFWPKLLMKVTDLRMIGACHASRFLHMKVECPTELFPPLFLEVFEDQEV
Opossum  (349) HYINHRKHNIPHFWPKLLMKVTDLRMIGACHASRFLHMKVECPTELFPPLFLEVFEDQEV
Platypus (349) HYINYRKHNIPHFWPKLLMKVTDLRTIGACHASRFLHMKVECPTELFPPLFLEVFEDQEV
Chicken  (349) HYINYRKHNIPHFWPKLLMKVTDLRMIGACHASRFLHMKVECPTELFPPLFLEVFEDQEV
Turtle   (349) HYINYRKHNIPHFWPKLLMKVTDLRMIGACHASRFLHMKVECPTELFPPLFLEVFEDQEV
```

Fig S3

B

TRα2 5'ss — Cryptic 5'ss

Species	Sequence
Human	ATGAAGGTGACTGACCTCCGCATGATCGGGGCCTGCCACGCCAGCCGCTTCCTCCACATGAAAGTCGAGTGCCCC
Rat	ATGAAGGTGACTGACCTCCGCATGATCGGGGCCTGCCACGCCAGCCGCTTCCTCCACATGAAAGTCGAGTGCCCC
Bat	ATGAAGGTGACTGACCTCCGCATGATCGGGGCCTGCCACGCCAGCCGCTTCCTCCACATGAAAGTCGAGTGCCCC
Dog	ATGAAGGTGACTGACCTCCGCATGATCGGGGCCTGCCACGCCAGCCGCTTCCTCCACATGAAAGTCGAGTGCCCC
Killer whale	ATGAAGGTGACTGACCTCCGCATGATCGGGGCCTGCCACGCCAGCCGCTTCCTCCACATGAAAGTCGAGTGCCCC
Cow	ATGAAGGTGACTGACCTCCGCATGATCGGGGCCTGCCACGCCAGCCGCTTCCTCCACATGAAAGTCGAGTGCCCC
Manatee	ATGAAGGTGACTGACCTCCGCATGATCGGGGCCTGCCACGCCAGCCGCTTCCTCCACATGAAAGTCGAGTGCCCC
Elephant shrew	ATGAAGGTGACTGACCTCCGCATGATCGGGGCCTGCCACGCCAGCCGCTTCCTCCACATGAAAGTCGAGTGCCCC
Opossum (M.dom)	ATGAAGGTGACTGACCTCCGGATGATAGGGGCCTGCCATGCCAGCCGCTTCTTACACATGAAAGTCGAGTGCCCC
Potoroo	ATGAAGGTGACTGACCTCCGGATGATCGGGGCCTGCCATGCCAGCCGGTTCCTACACATGAAAGTCGAGTGCCCC
Tasmanian devil	ATGAAGGTGACTGACCTCCGGATGATCGGGGCCTGCCATGCCAGCCGGTTCCTACACATGAAAGTCGAGTGCCCC
Platypus	ATGAAGGTGACAGATCTGCGCACGATCGGGGCCTGCCACGCCAGCCGCTTCCTGCACATGAAGGTGGAGTGCCCC
Chicken	ATGAAGGTGACGGACCTGCGCATGATCGGCGCCTGCCACGCCAGCCGCTTCCTGCACATGAAGGTGGAGTGCCCC
Ground tit	ATGAAGGTGACGGATCTGCGGATGATCGGCGCCTGCCACGCCAGCCGCTTCCTGCACATGAAGGTGGAGTGTCCC
Crow	ATGAAGGTGACGGATCTGCGGATGATCGGCGCCTGCCACGCCAGCCGCTTCCTGCACATGAAGGTGGAGTGTCCC
Turtle	ATGAAGGTGACCGACCTGCGCATGATCGGCGCCTGCCACGCCAGCCGCTTCCTGCACATGAAGGTGGAGTGCCCC
Lizard	ATGAAGGTGACCGACCTGCGCATGATTGGGGCTTGCCACGCCAGCCGCTTCCTGCACATGAAGGTGGAATGCCCC
Python	ATGAAGGTGACCGACCTACGCATGATTGGGGCTTGCCATGCCAGCCGCTTCCTGCACATGAAGGTGGAATGCCCC
Gekko	ATGAAGGTGACCGACCTGCGCATGATTGGGGCTTGCCACGCCAGTCGCTTCCTGCACATGAAGGTGGAATGCCCC
Aligator	ATGAAGGTGACCGACTTGCGCATGATTGGTGCCTGCCACGCCAGCCGCTTCCTGCACATGAAGGTGGAGTGCCCC
Frog	ATGAAGGTGACGGACCTGCGCATGATAGGGGCATGCCATGCCAGCCGCTTTCTGCACATGAAGGTTGAGTGCCCC
Axolotl	ATGAAGGTGACGGACCTGCGCATGATCGGGGCGTGCCACGCCAGCCGCTTCCTGCACATGAAGGTGGAATGCCCC
Zebrafish	ATGAAGGTGACGGACCTGCGGATGATCGGAGCCTGCCACGCCAGTCGCTTCCTGCACATGAAGGTGGAATGCCCC
Gar	ATGAAAGTGACGGACCTGCGGATGATCGGGGCCTGTCACGCCAGCCGCTTCCTGCACATGAAGGTGGAGTGTCCC
Coelacanth	ATGAAGGTAACAGACCTGCGGATGATCGGTGCATGCCACGCCAGCCGCTTCCTTCACATGAAGGTCGAGTGTCCC
Fugu	ATGAAGGTGACGGACCTCCGCATGATCGGAGCCTGCCACGCTAGCCGCTTCCTCCATATGAAGGTGGAATGTCCC
Pike	ATGAAGGTGACCGACCTGAGGATGATCGGCGCCTGCCACGCCAGCCGCTTCCTCCACATGAAGGTGGAGTGTCCC
	MetLysValThrAspLeuArgMetIleGlyAlaCysHisAlaSerArgPheLeuHisMetLysValGluCysPro

C

TRα1 (% amino acid identity)

	Platypus	Human	Rat	Opossum	Potoroo	Falcon	Tit	Turtle	Lizard
Human	92.9								
Rat	92.9	99.3							
Opossum (M.dom)	95.3	94.6	94.1						
Potoroo	95.6	94.4	93.9	99.8					
Falcon	93.6	90.4	90.9	94.6	94.9				
Ground tit	93.4	90.2	90.7	94.4	94.6	99.5			
Turtle	93.9	90.9	91.2	94.6	94.9	97.6	97.8		
Lizard	93.9	92.4	92.7	93.6	93.9	96.8	96.6	96.8	
Frog	90.0	88.2	88.2	90.9	90.7	91.4	91.2	91.9	90.7
