# Supplementary material for: Evolution of the Antisense Overlap between Genes for Thyroid Hormone Receptor and Rev-erbα and Characterization of an Exonic G-Rich Element That Regulates Splicing of TRα2 mRNA
Source: PLoS One. 2015 Sep 14;10(9):e0137893. doi: 10.1371/journal.pone.0137893 (PMC4569393; doi:10.1371/journal.pone.0137893)
Supplement: S4 Fig — (A) Alignment of 67 amino acids from the bidirectional coding sequence. X indicates a stop codon. Related species (reptiles/birds, marsupials and eutherian mammals) are grouped together and a consensus sequence is given for each group. Amino acids differing from the consensus are highlighted. Residues in the platypus sequence that differ from the eutherian consensus are also highlighted (B) Crosstable based on alignment in panel A giving the number of amino acid differences between the 67 codons corresponding to the BCS of TRα2. The following files were used for comparison of TRα2-related sequences: NP_003241.2 (human), NP_112396.2 (rat), NC_006591.3 (dog), XP_004378116.1 (Florida manatee), HM149331.1 (long nosed-potoroo), BK007078.1 (tammar wallaby), HM149332.1 (Virginia opossum), XM_001370259 (gray short-tailed opossum), NW_004929918 (Peregrine falcon), NW_005087648 (Tibetan ground-tit), NW_003338727.1 (green anole lizard), and NW_004848538.1 (western painted turtle). (PDF) [file pone.0137893.s004.pdf]

A

Sequences antisense to Rev-erbα exon 8 coding sequence:

```
Falcon            LLGVDAEGEQLLGVEVVEGAQVRQLQQQFGEARCLRRVRLQHEGAQGADQRLLQLLHRCRVLHARAV
Gound tit         LLGVDAEGEQLLGVEVVESAQVRQLQQQFGEARGLSRARLQHEGAQGPQQRLLQLLHXCRVLHARAV
Turtle            LLGVDAEGQQLLRMHVVQRAQVGQLQQQLGEARGLCGVVLEDQRAQGTDQGLLQLLHXRRVLHAGAV
Lizard            LLRINAEGEQFLRVHVVEGAQVRKLQQQFGEAGSLLGVILEDEGPKSPDXCLLQLLHXCLVLNARAI
Consensus         LLGVDAEGEQLL V VVE AQVRQLQQQFGEAR L  V L  EGAQG DQ LLQLLHX RVLHARAV

Opossum(Mdom)     SLGIYSEGQQLLGMHVVEGAQVRQLKQQLGEAGGLXGPILQDQGPKSPEQRLLQLFYRGRVLHTXSI
Opossum(Dvir)     SLCIYSEGQQLLGMHVVEGAQVRQLKQQLGEAGGLXGPILQDQRPKRPEQGLLQLFHRGRVLHTGSI
Potoroo           SLAIYSEGQQLLGMHVVEGTEVRQLKQXLGEAGCLXGPVLXDQCPKCSEQCLLQLFHRGXVLHTXSV
Wallaby           SLGIYSEGQQLLGMHVVEGTEVRQLKQXLGEAGSLXGPVLXDQCPKCSEQCLLQLFHRGRVLHTXSV
Consensus         SL IYSEGQQLLGMHVVEG   RQLKQ LGEAG LXGP L DQ PK  EQ LLQLFHRGRVLHTXS

Human TRα2        SLGVHPEGQQLLGMHVVQGPQVRQLEQQLGEAGSLQGPVLQHQSPKSPQQRLLELLHRSGILHARAV
Rat TRα2          SLGVHPEGQQLLGMHVVQGPQVRQLEQQFGEAGSLRGPVLQHQSPKSPQQRLLELLHRSGILHSRAV
Dog TRα2          SLGVHPEGQQLLGMHVVQGPQVRQLEQQLGEAGSLRGPVLQHQSPKSPQQRLLELLHRSGVLHARAV
Manatee TRα2      SLGVHPEGQQLLGMHVVQGPQVRQLEQQLGEAGSLQGPVLQHQSPKSPQQRLLELLHRSGILHARAV
Consensus         SLGVHPEGQQLLGMHVVQGPQVRQLEQQLGEAGSL GPVLQHQSPKSPQQRLLELLHRSGILHARAV

Platypus          SLGVHAEGQELLGMHVVQRAQIGQLEQQLGEAGRLGRAVLQHQGPQRPDQRLLQLLHRCHVLHARAV
```

B

Amino acid Differences Antisense to Exon 8 CDS

	Platypus	Human	Rat	M.dom.	D.vir.	Potoroo	Wallaby	Falcon	Tit	Turtle
Human	18									
Rat	20	3								
Opossum (M.dom)	25	21	22							
Opossum (D.vir)	26	23	24	7						
Potoroo	32	26	27	15	15					
Wallaby	29	22	23	11	12	5				
Falcon	22	27	26	28	30	36	32			
Ground tit	21	26	26	27	30	38	34	6		
turtle	21	26	28	27	25	33	28	20	21	
Lizard	31	30	30	27	27	32	30	23	25	28
