# Supplementary material for: Evolution of the Antisense Overlap between Genes for Thyroid Hormone Receptor and Rev-erbα and Characterization of an Exonic G-Rich Element That Regulates Splicing of TRα2 mRNA
Source: PLoS One. 2015 Sep 14;10(9):e0137893. doi: 10.1371/journal.pone.0137893 (PMC4569393; doi:10.1371/journal.pone.0137893)
Supplement: S5 Fig — (A) Clusters of three or more G or C residues are highlighted in red or green, respectively. The signal elements (PAS) for polyadenylation of TRα1 and Rev-erbα are boxed; arrow indicates position of the TRα2 splice site in rat and human. Sequences are referenced in S2–S4 Figs. (B) Comparison of the G-rich regions adjacent and antisense to the Rev-erbα stop codon (italicized) and the corresponding regions in marsupials, platypus, birds and turtle. Sequences are aligned at left to highlight similarities among diverse vertebrates. The 6 nt flanking regions at top left overlap the final 6 nt shown in panel A. (C) Conservation of sequence within diverse eutherian mammals. Sequences flanking the G30 region are indicated; the (antisense) stop codon for Rev-erbα is italicized and underlined. Two variant positions are indicated in the G30 sequence in pika and cow. Sequence files include pika (XM_004591164.1), dog (XM_003435250), ferret (XM_004764619.1), panda (XM_002924928), cow (NM_001046329.1), killer whale (XM_004282753.1), horse (XR_131463), star-nosed mole (XM_004684230.1), armadillo (NW_004465229), hedgehog tenrec (XR_193663.1), manatee (XM_004378060.1), bald eagle (XM_010585709.1), collared flycatcher (XM_005059802.1), and wallaby (BK007078) in addition to other Rev-erbα mRNAs in S2 Fig. (PDF) [file pone.0137893.s005.pdf]

**A**

**TRα1 PAS**

```
Rat                   AATAAATATATGATGAAAATAACCCCC--GGGCACCCCCCCTAGACTTGCGTGCTGTTTC
Human                 AATAAATATATGATGAAAATAACCCCCTTGGGCACCCCCCTTAGACTTGTGTGCTGTTTC
Opossum (M.dom.)      AATAAATATATGATGAAAATAACTCCCCTGAGAACCCTTCTGTGCCTGCATGGGCTATAT
Potoroo               AATAAATATATGATGAAAATAACTCCCTTGTGGACCCTTCTAAGCCTGCATCTGCTGTAT
Platypus              AATAAATATATGATGAAAATACCGAGGCGCACTGCCGCCCTCTACGTGTCTCCTGCCCCC
Ground tit            AATAAATATATGATGAAAAAAAAAACCAACCAACCAAACCCCACCCCACCCCCCGACCCA
Falcon                AATAAATATATGATGAAAAAAAAAACAAAAAAACCAACCAACCAAACCCAACCAAACCCA

Rat                   CCCATATT-----------CTCCCATCTGCTGGCAGAGTACCCACCCCACAAGCTGACCA
Human                 CCTATATC-----------CTCCCATCTGCTGGCAGAGTACCCACCCCACAAACTGACCA
Opossum (M.dom.)      TC---------------------CTTCCACCCCAAATCTGCCAGTAGCCTA---------
Potoroo               GTTGCCCC----------------TTCTACCCCAAATTTGCAGGTAGAGTA---------
Platypus              CGCCCCTTGGTCAGATCTTCCGCCTGCCGCCACTCCTGGCCAGGAGCTGCAGGGCGCCGG
Ground tit            CTCCCCAGCGCGG------CTGGATCCGGCCTCTGCGTCCCACCCCC----GGCCGCCAG
Falcon                CCCACACGGCTTGGCT-----GGATCCGGCCTCTTGCTTTCCACCACC---GGCCGGCCA

Rat                   GATGGAGA-------------------GGTGCCCCCCCAACCCCCAGCCTTGGCAGTAGT
Human                 GATGGAGA-------------------GGTGGCCCCC-----CCCAGCCTTGGCAGTATT
Opossum (M.dom.)      ------------------------------GGCTCTCGATCCTGCAGGAGGTCTCCTCTT
Potoroo               ------------------------------GGCCTTTGATCCAGAAGGCAGTATCCTCTT
Platypus              ACTGGGGAGGGCGGGGCGGGGGGAGGGGGTACCCCCTGTCCAGGTGGGCCGGGCAGCGCC
Ground tit            AACGGGGGTGTC-------GGGGTGAGGGGACCCCCCGGCGCTGCCAGCGGAGCCGT---
Falcon                AGAGGGGGGGGC-----GGGGGGGGGGAGGCCCCCCGGCACTGCCAGTGGAGCCGT---

Rat                   CCCCCCCCATTCCCCA-----------------------------AACGAGAGCACATCA
Human                 TCCACCCCACCCCCCCA-----------------------------AACGAGCACACACCA
Opossum (M.dom.)      CCCCCCACCCCC------------------------------------ACACACAGCA
Potoroo               CCCCCCACCTCT-----------------------------------AACACACACCA
Platypus              GGCTCACGTCTCCCCTCGGACCCTCCTCCCCCCGCCCCCGGCCGACCCGGGTACACACCA
Ground tit            ------------------------------------------------------------
Falcon                ------------------------------------------------------------
```

**PAS Rev-erbα major poly(A)**

```
Rat                   CAGAAGCCAGCTCAGCTG----TGAACTATTGGATTTGAGACAGGAACAGAACAAATCGA
Human                 CAGAAGCCAGCTCAGCTG----TGAACTATTGGATTTGAGACAGGAACAGAACAAATCAG
Opossum (M.dom.)      CAGAAGCCAGATCAGATAGATGTCAGCTACT-GATTTGAGATA-GAACAGGACACATCGG
Potoroo               CAGAAGCCATATCAGATAGATGTTAACTACT-GATTTGAGATGGGAACAGGACACATCAG
Platypus              CAGAAGGCAGAGC----AGACGTGAACGGGTGGATTTGAAATGGGCACAGAGCACACAGG
Ground tit            ------------------------------------------------------------
Falcon                ------------------------------------------------------------
```

# A (continued)

```
Rat                    GGGGCCAGAGGAGGGTGGGA---GAGCAAGAGTGGTTTAAATACGGGAGGAAGGGAGCA-
Human                  AGGGCCAGGGGAGGGTTGTGGGGGAGACAGAGTGGTTTAAATAGGGGAGGAGGGGAAGTT
Opossum (M.dom.)       GTGATGGTGGGTTCAACAGGAAGGGA TAGAGCAGTTC--CTTAAGGAGGGAACTATTT-
Potoroo                GTGAGG--GGGTTCAA-AGGAAGGAGAAAGAGCGGTTC--CATGGGAAGGGAGCTATTT-
Platypus               GGCTTGGGGAGGGAGATGACTTGGGGGGGGTGGGGACGGCGGACCGGGGGCGGGA----
Ground tit             GGGGGCGGTGCCCGATTTGGGGTGGGGGGTCCCAGC--ATCCTGGCGCCCA----CCTT-
Falcon                 GGGGCTGGTGCCCGACTGGGGTGGGAGGGGTGGGGGGTGTTCTGGCATCCTGGGGGGT

Rat                    ---TGGGGGTGGGGGGGAA-GAGTTATTTACAAGAAGGCTCAGGGGGCCAGA--------
Human                  CGGTGATGGGGGAGGGAGG-CAGGTATTTACAAGAAGGCTCAGGGGGCCAGA--------
Opossum (M.dom.)       ------------ACAAGAA-GGCTGGGCTGG---------AGGAGCCCAGA--------
Potoroo                ------------ACAAGAA-GGCTGGGGGTGGAGGTGGGAGTAGGGGGCAGA--------
Platypus               CGGCACTGCCGGCCTCGGCCCGGATTCCGGGGGGGCTGGGGCGGGGGGAGGTGTATTTAC
Ground tit             GGGGTGAATCCCTGCGAGTCCAACAGGCACCACGGGG----TTGGTGCTGCCCCGGGA-
Falcon                 GCAGCCCCCCCTGCCCCACCCTGGGGTGCCCGGCAGGCGCTGTGGGGGTTCGTGCTGCAC
```

**PAS Rev-erbα minor poly(A)**

```
Rat                    ------------------------GGCTC------ATCTTGGAATATTTTATAACAATAT
Human                  ------------------------GGCTC------ATCTTGGAATATTTTATAACAATAT
Opossum (M.dom.)       ------------------------GGCTC------TTCAGGGGATATTTTATAACAATAT
Potoroo                ------------------------GGCTC------ATCTTGGAATATTTTATAACAATAT
Platypus               ---AAGAAGGCTCCGATGTCCCGAGGCTCGTCCCTTCCGTGGAATATTTTATAACAATAT
Ground tit             GGGAGGGGGGAA-------GTCTGTGTGTCCCCCC--GCTGTGGTGTA-----------
Falcon                 CTGGGGGGGTTGGGTCGTGCGTCTGTGTCCCCTCCCCACCCGCGGTGCTA----------
```

**TRα2 3'ss**

```
Rat                    AAATAAGATTCTGG-------TTTGCTTTTCCTTTTCGTCTCGTAAAGGAGAGAGAAGTG
Human                  AAATAAGATTCTGG-------TTTGCTTTTCCTTTTCGTCTCGTAAAGGAGAGAGAAGTG
Opossum (M.dom.)       AAATAAGATTCTGGGGAA-GGGACGGTGTTTGTTTTTGTCTCTTAAAGAAGAGAGAAGTG
Potoroo                AAATAAGATCCTGGGGAAAGGGGTGGTGTTTGTTTTGGTCTCTTAAAGAAGAGA--AGTG
Platypus               AAATAAGAT---------------------------------------------------
Ground tit             ------------------------------------------------------------
Falcon                 ------------------------------------------------------------

Rat                    CAGAGTTCGATTC TGTACA (G30)
Human                  CAGAGTTCGATTC TGTACA
Opossum (M.dom.)       CAGAGTTCAGTTC TGTACA
Potoroo                CAGAGTTCAGTTC TGTACA
Platypus               ------------- CCGGCA
Ground tit             ------------- TGTACA
Falcon                 ------------- TGTACA
```

# B

## G30-like Sequences of mammals, birds and reptiles

```
                                                                              Rev-erbα stop
Rat(eutherian    TGTACA AGGGGG                  CAGCGGCAGAAGGCCGGCCGGGCGGG TCA CTGGG
    consensus)
Opossum(Mdom)    TGTACA AGGACAG     GGCCCGATGGAGACAGAGATCGGTGGAGAGGCTGTAG TCA CTGGG
Opossum(Dvir)    TGTACA AGGACAG     GGCCCGATGGAGGCAGAAATTGGTGGGGGGCTGTAG TCA CTGGG
Potoroo          TGTACA AGGAGAG     AGCTTGATGGAGACAGCAGGGGATGGGGAGGCTGAGG TCA CTGGC
Wallaby          TGTACA AGGAGAG     AGCTTGATGGAGACAGCAGGGGATGGGGAGGCTGAGG TCA CTGGC

Platypus         CCGGCA AGGAGAG  CAGGGGGGGCGGGCCGGGGGAGGAGGGGGAAGGGGTGGGC TCA CTGGG

Ground Tit       TGTACA        GGGGTGCGCGGGGGGGTTCCGGGGGGGGGGGTCCGGGGGGCA CTA CTGGG
Falcon           TGTACA GGGGTGGCGGGTGCGTGGGGGAGGGGG TCCCTGGGGTCCGTCGGGGCC CTA CTGGG
Bald Eagle       TGTACA AGGGTGGTGGGTGCGCGGGGGGG     TCCCTGGGGTCTGTGGGGGCC CTA CTGGG
Flycatcher       TGTACA        GGGGTGCGCGGGGGGG      CCC         GGGGGGCA CTA CTGGG

Turtle           AGCAAT AGAGTCCGGTGGGGTTTCTCTGCGGAGGGTCCCGGGGT GGGGGGAGCC CTA CTGGG
```

# C

## Eutherian G30 sequences

```
                                                      Rev-erbα stop
Human              TGTACA AGGGGGCAGCGGCAGAAGGCCGGCCGGGCGGG TCA CTGGG
Rat                TGTACA AGGGGGCAGCGGCAGAAGGCCGGCCGGGCGGG TCA CTGGG
Pika               TGTACA AGGGGGCAGCGGCGGCAGGCCGGCCGGGCGGG TCA CTGGG
Dog                TGTACA AGGGGGCAGCGGCAGAAGGCCGGCCGGGCGGG TCA CTGGG
Ferret             TGTACA AGGGGGCAGCGGCAGAAGGCCGGCCGGGCGGG TCA CTGGG
Panda              TGTACA AGGGGGCAGCGGCAGAAGGCCGGCCGGGCGGG TCA CTGGG
Cow                TGTACA AGGGGGCAGCGGCGGAAGGCCGGCCGGGCGGG TCA CTGGG
Killer whale       TGTACA AGGGGGCAGCGGCAGAAGGCCGGCCGGGCGGG TCA CTGGG
Horse              TGTACA AGGGGGCAGCGGCAGAAGGCCGGCCGGGCGGG TCA CTGGG
Star-nose mole     TGTACA AGGGGGCAGCGGCAGAAGGCCGGCCGGGCGGG TCA CTGGG
Armadillo          TGTACA AGGGGGCAGCGGCAGAAGGCCGGCCGGGCGGG TCA CTGGG
Manatee            TGTACA AGGGGGCAGCGGCAGAAGGCCGGCCGGGCGGG TCA CTGGG
Hedgehog tenrec    TGTACA AGGGGGCAGCGGCAGAAGGCCGGCCGGGCGGG TCA CTGGG
```
