# Supplementary figures and images for: Evolution of the Antisense Overlap between Genes for Thyroid Hormone Receptor and Rev-erbα and Characterization of an Exonic G-Rich Element That Regulates Splicing of TRα2 mRNA
Source: PLoS One. 2015 Sep 14;10(9):e0137893. doi: 10.1371/journal.pone.0137893 (PMC4569393; doi:10.1371/journal.pone.0137893)

**Number of EST Sequences**

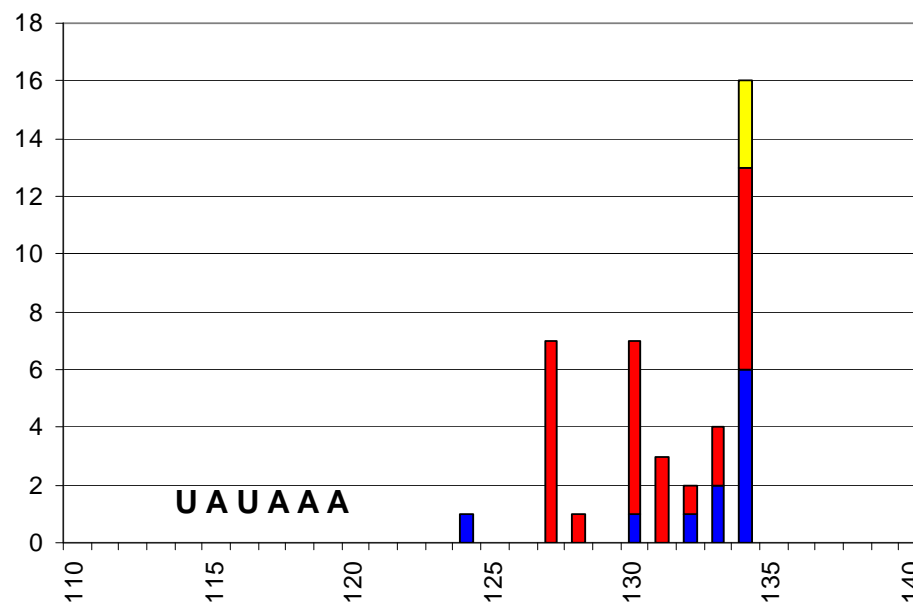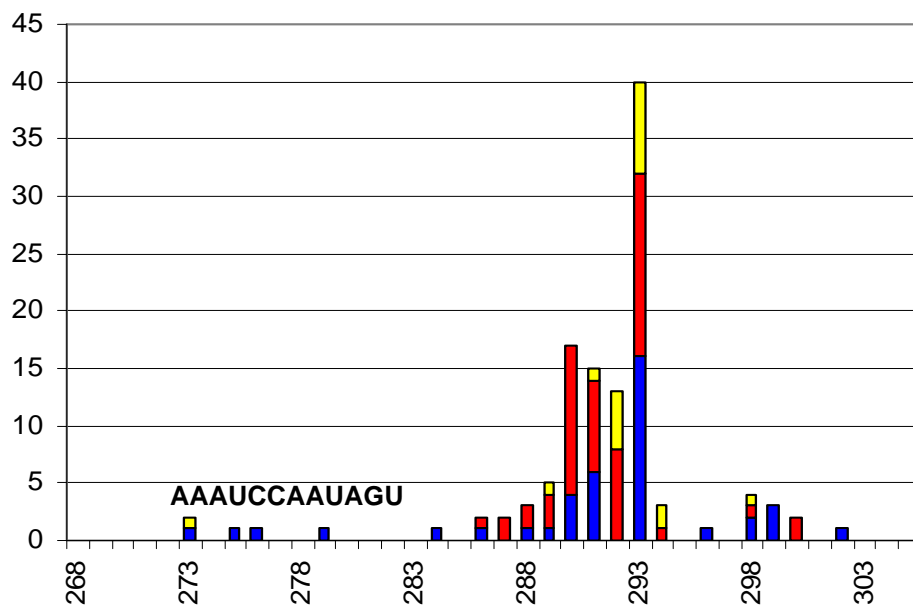

**Distance from Rev-erb $\alpha$  CDS**

Supplement: S6 Fig — ESTs were sorted according to position of poly(A) sites. These formed two clusters representing the minor upstream poly(A) site (upper chart) and the major downstream site (lower chart). Positions are given relative to the rat Rev-erbα coding sequence (cds) and to the conserved PAS sites for each poly(A) site. Note different scales for each chart. (PDF) [file pone.0137893.s006.pdf]

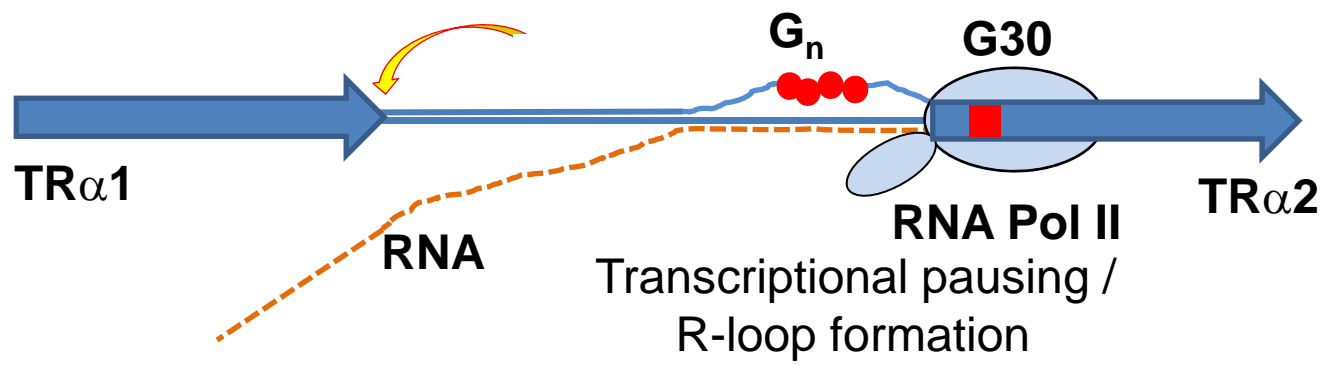

Supplement: S8 Fig — G-clusters within the G30 region (red box) and within the intron (red circles) may promote RNA polymerase pausing (light blue ellipses) and R-loop formation in a transcription-dependent manner as represented schematically by parallel dotted orange (RNA) and blue (DNA) lines within intron. Pausing near the G30 element, near the 3’ ss may inhibit TRα2 splicing and promotes TRα1 polyadenylation as indicated by curved arrow. Large blue arrows represent 3’ exons and poly(A) sites for TRα1 and TRα2 mRNAs. The effect of G-clusters on pausing and R-loop formation may involve G-quadruplex structure on either the displaced strand of DNA (irregular blue line) or the RNA transcript. (PDF) [file pone.0137893.s008.pdf]
