# Supplementary material for: Evolution of the Antisense Overlap between Genes for Thyroid Hormone Receptor and Rev-erbα and Characterization of an Exonic G-Rich Element That Regulates Splicing of TRα2 mRNA
Source: PLoS One. 2015 Sep 14;10(9):e0137893. doi: 10.1371/journal.pone.0137893 (PMC4569393; doi:10.1371/journal.pone.0137893)
Supplement: S7 Fig — (A) Diagram showing relative positions of nested primers (A-D) as in Fig 3A. (B) Gel electrophoresis of 3’RACE products of platypus liver RNA following reverse transcription and multiple rounds of 3’RACE PCR amplification. Lane 1: first round 3’RACE with upstream primer A; lanes 2–5 second round 3’ RACE PCR using product shown in lane 1 with primers B, A, C and D, respectively. Products from lanes 2 and 5 were sequenced and correspond to polyadenylation at the minor and major poly(A) sites, respectively. Lane 6 shows the third round PCR product obtained by amplifying primer C 3’RACE product (from lane 4) with primer D. This product when sequenced identified a downstream polyadenylation site. Its anomalous size reflects mispriming within the Cn region. Products from isolated bands in lanes 2 and 6 were sequenced both directly and after cloning. Band from lane 5 was sequenced after cloning. Asterisks (*) indicate PCR reactions subjected to second (lane 1) and third (lane 4) rounds of amplification. Arrows indicate bands sequenced. (C) Sequence of Rev-erbα 3’ UTR in platypus. The positions of the four poly(A) sites are indicated (/). The upstream PAS and AT-rich sequences are underlined, with the TRα1 PAS on the opposite strand indicate by the dotted line. (PDF) [file pone.0137893.s007.pdf]

A

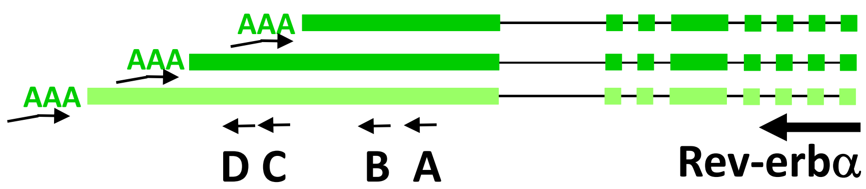

B

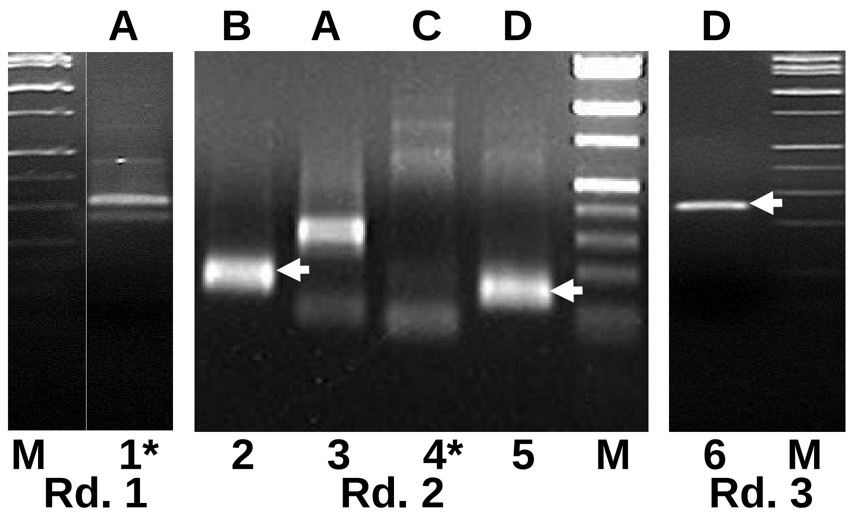

C

```
                               Rev-erbα stop
2314                                   TGA GCCC ACCCCTTCCC CCTCCTCCCC
2341 CGGCCCGCCC CCCCTGCTCT CCTTGCCGGA TCTTATTTAT ATTGTTATAA AATATTCCAC
          Poly(A)site
2401 GGAAGGG/ACG AGCCTCGGGA CATCGGAGCC TTCTTGTAAA TACACCTCCC CCCGCCCCAG
2461 CCCCCCCGGA  ATCCGGGCCG AGGCCGGCAG TGCCGTCCCG CCCCCGGTCC GCCGTCCCCA
2521 CCCCCCCCCA  AGTCATCTCC CTCCCCAAGC CCCTGTGTGC TCTGTGCCCA TTTCAAATCC
             Alternative poly(A) site
2581 ACCCGTTCAC GTC/TGCTCTG CCTTCTGTGG TGTGTACCCG GGTCGGCCGG GGGCGGGGGG
2641 AGGAGGGTCC GAGGGGAGAC  GTGAGCCGGC GCTGCCCGGC CCACCTGGAC AGGGGGTACC
2701 CCCTCCCCCC GCCCCGCCCT  CCCCAGTCCG GCGCCCTGCA GCTCCTGGCC AGGAGTGGCG
2761 GCAGGCGGAA GATCTGACCA  AGGGGCGGGG GGCAGGAGAC ACGTAGAGGG CGGCAGTGCG
                                                    Alternative poly(A) site
2821 CCTCGGTATT TTCATCATAT  ATTTATTACA TAAATATATA GTAAAATAGA CAAGC/AACAA
    Alternative poly(A) site
2881 TT/ACCATAAA AATATCTTTC TTTAAAATCC
```
